# JoGo 1.0: the ACTG hierarchical nomenclature and database covering 4.7 million haplotypes across 19,194 human genes

**DOI:** 10.1093/nar/gkaf1232

**Published:** 2025-11-29

**Authors:** Masao Nagasaki, Toshiaki Katayama, Yuki Moriya, Yayoi Sekiya, Shuichi Kawashima, Ryo Teraoka, Shuto Machida, Taichi Matsubara, Hiroki Hashimoto, Akihiro Asakura, Akio Nagano, Riu Yamashita, Toyoyuki Takada, Nobutaka Mitsuhashi, Mayumi Kamada, Yasuyuki Ohkawa, Katsushi Tokunaga, Yosuke Kawai, Variant Information Standardization Collegium, Masao Nagasaki, Toshiaki Katayama, Yuki Moriya, Shuichi Kawashima, Shuto Machida, Taichi Matsubara, Akio Nagano, Riu Yamashita, Toyoyuki Takada, Nobutaka Mitsuhashi, Takatomo Fujisawa, Mayumi Kamada, Daisuke Satoh, Tsuyoshi Hachiya, Masaki Ohira, Yoko Kuroki, Patricia Severino, Samuel Papa Kwesi Owusu, Yifan Huangfu, Atsushi Doi, Yuichiro Hara, Minae Kawashima, Yosuke Kawai

**Affiliations:** Medical Research Center for High Depth Omics, Medical Institute of Bioregulation, Kyushu University, Fukuoka 812-8582, Japan; Department of Center for Genomic Medicine, Graduate School of Medicine, Kyoto University, Kyoto 606-8507, Japan; Database Center for Life Science, Joint Support-Center for Data Science Research, Research Organization of Information and Systems, 178-4-4 Wakashiba, Kashiwa, Chiba 277-0871, Japan; Database Center for Life Science, Joint Support-Center for Data Science Research, Research Organization of Information and Systems, 178-4-4 Wakashiba, Kashiwa, Chiba 277-0871, Japan; Medical Research Center for High Depth Omics, Medical Institute of Bioregulation, Kyushu University, Fukuoka 812-8582, Japan; Database Center for Life Science, Joint Support-Center for Data Science Research, Research Organization of Information and Systems, 178-4-4 Wakashiba, Kashiwa, Chiba 277-0871, Japan; Medical Research Center for High Depth Omics, Medical Institute of Bioregulation, Kyushu University, Fukuoka 812-8582, Japan; Medical Research Center for High Depth Omics, Medical Institute of Bioregulation, Kyushu University, Fukuoka 812-8582, Japan; Medical Research Center for High Depth Omics, Medical Institute of Bioregulation, Kyushu University, Fukuoka 812-8582, Japan; Medical Research Center for High Depth Omics, Medical Institute of Bioregulation, Kyushu University, Fukuoka 812-8582, Japan; Medical Research Center for High Depth Omics, Medical Institute of Bioregulation, Kyushu University, Fukuoka 812-8582, Japan; PENQE Inc., 2-20-11 Kojima, Taito-ku, Tokyo 111-0056, Japan; Division of Translational Informatics, Exploratory Oncology Research & Clinical Trial Center, National Cancer Center, 6-5-1 Kashiwanoha, Kashiwa, Chiba 277-8577, Japan; Department of Computational Biology and Medical Sciences, Graduate School of Frontier Sciences, The University of Tokyo, 5-1-5 Kashiwanoha, Kashiwa, Chiba 277-8561, Japan; Integrated Bioresource Information Division, RIKEN BioResource Research Center, 3-1-1 Koyadai, Tsukuba, Ibaraki 305-0074, Japan; Database Center for Life Science, Joint Support-Center for Data Science Research, Research Organization of Information and Systems, 178-4-4 Wakashiba, Kashiwa, Chiba 277-0871, Japan; Department of Data Science, School of Frontier Engineering, Kitasato University, 1-15-1, Kitazato, Minami, Sagamihara, Kanagawa 252-0373, Japan; Medical Research Center for High Depth Omics, Medical Institute of Bioregulation, Kyushu University, Fukuoka 812-8582, Japan; Genome Medical Science Project, National Institute of Global Health and Medicine, Japan Institute for Health and Security, Shinjuku-ku, Tokyo 162-8655, Japan; Genome Medical Science Project, National Institute of Global Health and Medicine, Japan Institute for Health and Security, Shinjuku-ku, Tokyo 162-8655, Japan; Bioinformation and DDBJ Center, Research Organization of Information and Systems, National Institute of Genetics, 1111 Yata, Mishima-shi, Shizuoka 411-8540, Japan

## Abstract

The Joint Open Genome and Omics Platform 1.0 (JoGo) is a global, long-read-based human haplotype database covering 19 194 MANE-standardized protein-coding genes. JoGo introduces a novel ACTG hierarchical nomenclature—A (amino acid), C (coding), T (transcript), and G (gene body)—that assigns numeric identifiers in descending order of global frequency. Using high-fidelity long-read sequencing, we assembled haplotype-resolved contigs for 258 globally sampled genomes, including 108 sequenced in-house. We cataloged 174 376 A-, 300 610 C-, 486 288 T-, and 3 695 204 G-level haplotypes (4 656 478 in total). Haplotype IDs are assigned once globally across all sequences, including those originating from GRCh38 and CHM13v2 reference assemblies, embedding reference haplotypes within the same frequency-ranked space and enabling direct cross-assembly comparison. JoGo maps functional variants from ClinVar, GWAS Catalog, and GTEx onto their corresponding ACTG-haplotypes and provides haplotype-expression QTL results from 1280 HapMap RNA-seq samples across three independent studies. The web portal provides flexible search by gene name, variant ID, or ACTG code. It offers both an interactive online viewer and a privacy-preserving local viewer for secure integration with user data. JoGo enables high-resolution exploration of haplotype diversity, facilitating the identification of functional variants relevant to gene regulation, disease associations, and precision medicine. JoGo 1.0 is freely accessible at https://jogo.csml.org.

## Graphical Abstract

**Graphical Abstract:**
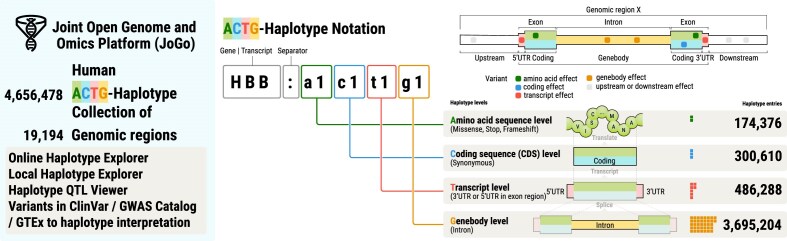

## Introduction

Haplotype-level genomic analysis offers a powerful approach for understanding the functional consequences of genetic variation by capturing combinations of linked variants and evaluating their collective effects on gene regulation [1, 2], splicing, and transcription [3]. To date, such analyses have been developed for a limited set of complex gene families—most notably the human leukocyte antigen (HLA), killer immunoglobulin-like receptor [4], and cytochrome P450 loci [5].

High-resolution haplotype catalogs for these loci have demonstrated substantial value in both clinical and research contexts, including transplant matching [6], identifying alleles protective against viral infection [7], stratifying autoimmune disease risk [8], optimizing drug dosing [9], fine-mapping expression quantitative trait loci (eQTLs) [10], and interpreting regulatory or structural variants missed by single-variant genome-wide association studies [11, 12].

However, extending haplotype-based approaches genome-wide has been hindered by technical limitations. Statistical phasing from short-read data is prone to switch errors, particularly in long or structurally complex regions, which reduces accuracy for full-length haplotype reconstruction [13, 14]. As a result, most human genes still lack comprehensive haplotype definitions that integrate all linked variants into biologically coherent units.

Recent advances in third-generation long-read sequencing—particularly high-fidelity (HiFi) and nanopore technologies—along with improved assembly and variant annotation tools [15–18], now enable accurate, haplotype-resolved assemblies across entire gene bodies from single molecules. These capabilities make it feasible to construct population-scale haplotype databases that extend beyond a few immunogenetic or pharmacogenomic loci to encompass thousands of protein-coding genes.

Here, we present the Joint Open Genome and Omics Platform Version 1.0 (JoGo 1.0; https://jogo.csml.org), the first genome-wide, long-read-based human haplotype database. JoGo 1.0 catalogs ~4.7 million haplotypes for 19 194 MANE-standardized protein-coding genes [19], derived from 258 globally sampled genomes—including 108 sequenced in-house using HiFi technology—and includes haplotypes from both GRCh38 and CHM13v2 within a single global frequency ranking. Unlike earlier resources limited to a few loci or a single reference coordinate system, JoGo unifies population genomes and major reference assemblies under one ACTG hierarchical nomenclature—A (amino acid), C (coding sequence), T (transcript), and G (gene body)—assigning numeric identifiers in descending order of global frequency (e.g. a1 and c2). Inspired by the HLA allele naming convention but generalized genome-wide, this framework supports cross-gene comparison, population diversity analysis, and direct integration with external resources such as ClinVar, the GWAS Catalog, and GTEx [20–22]. In parallel, JoGo offers an interactive online explorer and a privacy-preserving local viewer that enable the analysis of sensitive datasets alongside JoGo references without requiring cloud upload.

The availability of full-length haplotype assemblies enables systematic evaluation of variant combinations—including long structural variations such as Alu and LINE insertions—and their functional impacts, e.g. coordinated transcription factor binding [23], cooperative regulation via 3′ and 5′ UTR variants [24], and splicing differences [3]. Detailed descriptions of data content, interface features, and haplotype-level eQTL analysis (pairwise haplotype–expression association testing; hereafter “haplotype QTL”) are provided in the following sections.

## Overview of JoGo portal and ACTG-haplotype nomenclature

### Overview of JoGo portal

**Figure 1.**
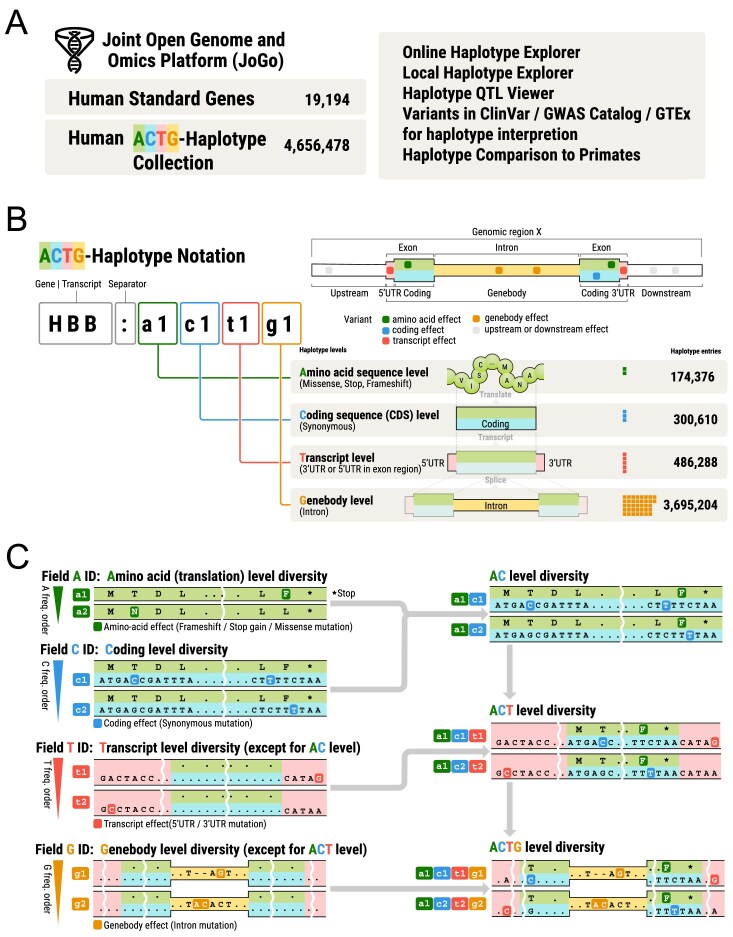
(**A**) Overview of JoGo portal contents and functions. (**B**) Overview of the ACTG-haplotype notation and the haplotype collections in the JoGo database. (**C**) Example of A-, C-, T-, and G-level haplotype ID assignment and hierarchical haplotype ID construction.

JoGo 1.0 catalogs 4 656 478 ACTG-haplotypes for 19 194 MANE-standardized human genes, derived from 258 globally sampled genomes across five continental superpopulations (Supplementary Tables S1 and S2), and makes them accessible through both online and local haplotype explorers (Figs 1A, 3, and 4). The resource combines 108 newly generated high-fidelity long-read genomes with publicly available datasets, providing a broad foundation for high-resolution haplotype analysis. To bridge traditional variant-centric analyses with haplotype-level insights, JoGo links each haplotype to clinical annotations (ClinVar) [22], phenotype associations (GWAS Catalog) [20], and expression correlations across multiple tissues (GTEx) [21]. To strengthen direct connections between haplotype structure and gene expression, JoGo also serves haplotype QTL results generated from 1280 RNA-Seq samples in three independent lymphoblastoid cell line panels, with association testing performed across all ACTG hierarchical levels [25–27]. Additionally, users can compare human haplotypes with primate reference genomes to interpret sequence and structural variants in an evolutionary context. Detailed descriptions of these portal features follow in later sections.

To contextualize JoGo’s scope and unique ACTG framework, we contrast it with IPD-IMGT/HLA (official HLA allele repository) [4] and PharmVar (locus-specific pharmacogene star alleles) [5] (Supplementary Table S3).

## Concept of the ACTG-haplotype nomenclature in JoGo

### Coding gene

Figure 1B illustrates the ACTG-haplotype nomenclature introduced in JoGo, using the beta-globin gene (*HBB*) as an example. This hierarchical system partitions each gene into coding sequence regions (A and C), exonic non-coding regions (T), and intronic regions (G). The A-level captures amino acid variation (missense mutation and in-frame insertion and deletion) in the protein product (e.g. a1), the C-level captures synonymous mutation within the coding sequence (e.g. c1), the T-level captures differences confined to exonic non-coding sequence (namely the 5′ and 3′ UTRs in coding genes) (e.g. t1), and the G-level captures intronic diversity within the gene body (e.g. g1; when a transcript is intronless, g0 is used). Two optional fields, U and D, record variants located 5 kb upstream and downstream of the gene, respectively. Within each level, JoGo assigns numeric identifiers in descending order of global frequency; for example, a1 is at least as common as a2. Concatenating the level-specific IDs without delimiters yields multilayer haplotype codes: combining A with C produces an AC code (e.g. HBB:a1c1), adding T yields an ACT code (e.g. HBB:a1c1t1) that spans the full transcript, and including G yields an ACTG code (e.g. HBB:a1c1t1g1) representing the entire gene body. A value of zero denotes a non-applicable level, such as a0c0 for a non-coding gene or a0c1 for a coding haplotype lacking a translated product due to a start-loss or stop-gain variant. To distinguish multiple transcripts for a single gene, the transcript ID can be included (e.g. NM_000518:a1c1 and ENST00000335295:a1c1). The ACTG-haplotype version is indicated by appending the version number as metadata, separated by a colon (e.g. HBB:a1c1:v1 for JoGo 1.0).

### Example of the ACTG-haplotype annotation

Figure 1C provides an example of how the ACTG-haplotype notation is applied in JoGo, illustrating the hierarchical relationships among the four individual levels (A, C, T, G) and their combinations (AC, ACT, ACTG). In this example, an amino acid substitution first divides the locus into two A-level haplotypes, a1 and a2, with a1 being at least as common as a2. Within the common a1 background, two synonymous DNA variants define C-level haplotypes c1 and c2, again ranked by global frequency (c1 ≥ c2). UTR polymorphisms further differentiate T-level haplotypes t1 and t2, with t1 occurring more frequently than t2. Additionally, intronic variants distinguish G-level haplotypes g1 and g2, with g1 having the higher frequency. The resulting codes—such as a1c1, a1c1t1, and a1c1t1g1 on one branch, and a1c2, a1c2t2, and a1c2t2g2 on the other—demonstrate how the ACTG concatenation progressively expands the sequence scope from protein-coding changes to the whole gene body, while preserving the frequency-based ordering at each hierarchical level.

### Non-coding gene

Non-coding transcripts, (e.g. lincRNAs, antisense lncRNAs, miRNA host genes, snoRNA/snRNA host genes, and transcribed processed pseudogenes) are represented with a TG-only scheme in which A = C = 0. The T-level indexes exonic non-coding sequence (i.e. transcript exons of non-coding genes), and the G-level indexes introns (if a transcript is intronless, g0 is used). As with coding genes, identifiers within each level are assigned in descending global frequency. When multiple transcripts exist for a non-coding gene, a transcript identifier can be prepended to disambiguate haplotypes. The lincRNA *MALAT1* has several isoforms, including NR_002819 and NR_144568 in RefSeq database [28]. They can be represented as NR_002819:a0c0t1g0 (short form: NR_002819:t1g0) and NR_144568:a0c0t1g1 (NR_144568:t1g1), respectively.

### Regulatory elements in intergenic regions

To annotate regulatory regions external to the gene body (e.g. enhancers, silencers, and CTCF-bound insulators), we introduce the R-level (Regulatory), which is functionally separated from the G-level. Each regulatory element is anchored by a RegionID that deterministically yields a sequence on a specified reference assembly: either an external registry accession (e.g. ENCODE cCRE EH38E3212459, corresponding to GRCh38 chr17:17 973 344–17 973 515) [29] or a reference-anchored interval identifier (e.g. GRCh38_chr17_17973344_17973515). Using the RegionID as a prefix, we assign frequency-ranked r-IDs within that region (e.g. EH38E3212459:r1, EH38E3212459:r2; GRCh38_chr17_17973344–17973515:r1, GRCh38_chr17_17973344–17973515:r2), where r1 ≥ r2 in global frequency.

## Materials and methods

Data retrieval and processing to construct the ACTG-haplotype dataset

## Data resource

We constructed the JoGo 1.0 dataset from a combination of newly generated and publicly available high-fidelity long-read sequencing data. A total of 108 genomes were sequenced in-house from lymphoblastoid cell lines (LCLs) derived from individuals previously included in the 1000 Genomes Project [30], representing five continental superpopulations (Supplementary Tables S1 and S2). LCLs were obtained from the NHGRI Sample Repository for Human Genetic Research via the Coriell Institute for Medical Research, with DNA extracted using the Nanobind CBB Big DNA Kit (Pacific Biosciences) or, in some cases, provided directly as purified DNA by Coriell. Sequencing was performed on the Sequel IIe or Revio platforms (Pacific Biosciences) using HiFi protocol.

In addition to these 108 genomes (PRJDB18487), we incorporated 10, 29, 17, and 94 publicly available HiFi datasets from JaSaPaGe (PRJDB19680) [31], the Human Pangenome Reference Consortium (HPRC) release 1, HPRC Plus, and part of the forthcoming HPRC next release candidate (PRJNA731524, PRJNA701308), respectively (Supplementary Table S2) [32]. In total, JoGo 1.0 comprises 258 genomes sampled across five continental superpopulations—East Asia (130; EAS), Africa (50; AFR), America (44; AMR), South Asia (25; SAS), and Europe (9; EUR) (Supplementary Table S1)—providing a globally representative foundation for haplotype cataloguing.

## Construction of ACTG-haplotype resource from long-read sequencing datasets

### Read alignment and assembly

Raw HiFi reads from 258 datasets were aligned to the GRCh38 human reference genome (ALT contigs excluded) using pbmm2 (v1.10.0), and multiple sequencing runs per sample were merged with SAMtools (v1.12) [33]. For each of the 19 316 MANE v1.2 gene regions, reads spanning ±5 kb from transcription start and end sites were extracted and locally assembled with hifiasm (v0.16.1) [15]. Diploid assemblies were retained only when two high-quality, full-length contigs—including the ±5 kb flanking regions—were recovered for autosomal loci or chrX in females. For chrX and chrY in males, haploid assemblies were accepted if a single contig fully spanned the target (end-to-end coverage). Assemblies producing multiple or incomplete contigs were excluded from further analysis.

### Transcript alignment to human and primate genomes

MANE transcript sequences (v1.2) were aligned to CHM13v2 [34] and four telomere-to-telomere (T2T) primate reference genomes—chimpanzee, bonobo, gorilla, and orangutan [35, 36]—using Minimap2 (v2.23, splice:hq mode) [37]. For each aligned locus, we extracted ±7 kb to encompass the upstream (U) and downstream (D) regions, which often contain structural variation, and defined syntenic intervals. These intervals were then mapped back to GRCh38 with Minimap2 in HiFi DNA alignment mode, providing a cross-species anchor for JoGo gene definitions on both reference assemblies.

### ACTG-haplotype nomenclature and construction

For each gene region, haplotype-resolved contigs were parsed to generate a “haplotype BAM,” retaining alignments trimmed to ±5 kb from the gene boundaries. Variants were called from alignment CIGAR strings and annotated with SnpEff (v5.2c) [38] using MANE transcript models. To minimize artifacts, especially in homopolymer-rich coding regions, we applied context-specific filtering of frameshift indels based on empirically calibrated per-contig indel quality values (QV) for each homopolymer length and base (Supplementary Table S4). For each candidate variant, the lower of the reference- and alternate-context QVs was converted to a per-contig error probability, and a binomial model was used to compute the probability of observing the supporting allele count by chance. This probability was then transformed into a variant-level quality value (VQV). Variants were retained only if VQV ≥ 100 for A- and C-level haplotypes (error probability ≤ 10⁻^10^, ideally yielding fewer than one expected false base call across all coding positions) or VQV ≥ 50 for T- and G-level haplotypes (error probability ≤ 10⁻⁵; Supplementary Note).

After this contig-cleaning step, each assembled contig was translated into four hierarchical haplotype levels: A, C, T, and G. Identical sequences within each level were clustered and assigned a unique numeric identifier, starting at 1, with lower numbers indicating higher global frequency (e.g. a1 ≥ a2). When the relevant sequence was absent—because the coding region was disrupted (e.g. a stop-loss variant) or the locus lacked that segment altogether (e.g. genes without 5′ and 3′ UTRs in the annotation)—the identifier zero was assigned.

## Results

### ACTG-haplotype catalog summary

JoGo 1.0, leveraging high-fidelity long-reads, assembled full-length, phased haplotypes and, after applying per-gene end-to-end coverage (as defined in the “Materials and methods” section) and variant quality criteria, retained 19 194 MANE v1.2 genes (18 319 autosomal, 831 X-linked, and 44 Y-linked) passed both coverage and quality filters, yielding 174 376 A-, 300 610 C-, 486 288 T-, and 3 695 204 G-level haplotypes across the 258 genomes analyzed (Fig. 1B; Supplementary Table S5). For each gene, the number of haplotypes at every ACTG level is listed (Supplementary Table S5), and the across-gene distributions of these counts are shown (Supplementary Fig. S1).

**Figure 2.**
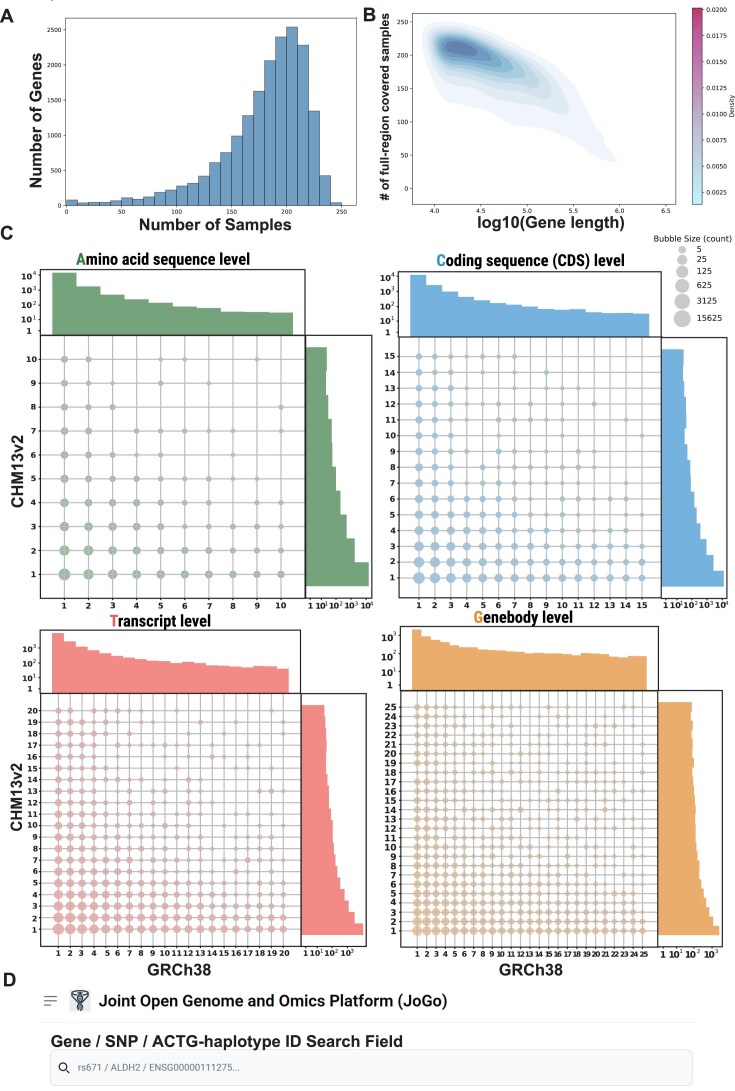
(**A**) Histogram of gene coverage by number of haplotype-resolved samples in JoGo 1.0. The *X*-axis represents the number of samples (out of 258 total) in which both haplotypes span the entire gene, and the *Y*-axis indicates the number of genes that achieve this level of sample coverage. (**B**) Density plot of gene length versus sample coverage in JoGo 1.0. The *X*-axis represents gene length on a log_10_ scale (bp), and the *Y*-axis shows the number of entirely spanned, haplotype-resolved samples (out of 258) per gene. A negative trend indicates that longer genes are resolved in fewer samples. (**C**) Bubble-grid and marginal histograms of global ACTG-haplotype ID ranks on GRCh38 versus CHM13v2 for each haplotype level (A, C, T, G). In the central grid, the *X*-axis denotes the ID rank on GRCh38, the *Y*-axis the rank on CHM13v2, and the bubble size is proportional to the number of genes with that rank pairing. The histograms along the top (GRCh38) and right (CHM13v2) margins show the overall frequency distribution of ID ranks on each reference. (**D**) Screenshot of the JoGo 1.0 portal’s top-page search interface, demonstrating support for queries by gene name, gene symbol, Ensembl ID, RefSeq ID, dbSNP rsID, or hierarchical ACTG-haplotype notation (e.g. ALDH2:a1c1t1g1).

For autosomal genes, the median number of entirely spanned, haplotype-resolved samples was 189 (mode 210). In total, 93.9% of autosomal genes and 94.6% of X-linked genes were covered in at least 100 individuals (Fig. 2A; Supplementary Tables S6–S8). Coverage of Y-linked genes reached 50%, reflecting the smaller number of male samples (*n* = 132; Supplementary Table S9). As expected, gene length showed a negative correlation with sample coverage (*R*^2^ = 0.414; β = –63.1 bp per sample; Fig. 2B).

### Gene length and haplotype diversity

To control for unequal sample sizes across genes, we restricted subsequent analyses to the 18 095 genes with ≥200 available haploids and performed random subsampling to 200 haploids per gene (100 replicates). For each gene and for each level (A, C, T, and G), we examined the relationship between the median number of distinct haplotypes (per level) and the corresponding level-specific sequence length (bp) (Supplementary Table S10). As expected, haplotype count was positively correlated with length: A (*R*^2^ = 0.39; β = 122 bp per distinct haplotype), C (*R*^2^ = 0.44; β = 84 bp), T (*R*^2^ = 0.62; β = 76 bp), and G (*R*^2^ = 0.35; β = 532 bp) (Supplementary Table S11; Supplementary Fig. S2).

The few loci with limited haplotype coverage overlap highly duplicated or structurally complex families (e.g. *USP17L, FAM90A, DEFB* clusters, *TP53TG3, TSPY*). Because read retrieval and local assemblies were anchored to GRCh38 MANE windows (ALT contigs excluded) and we retained only both-haplotype, end-to-end assemblies, these deficits are consistent with incomplete GRCh38 reference representation (e.g. collapsed or gapped segments and paralog conflation) rather than with limitations of the assembly procedure itself [39, 40].

The JoGo portal allows users to examine per-gene haplotype diversity metrics and to identify the most and least diverse loci using top-page filters and dedicated statistics pages (Supplementary Figs S3 and S4).

### Low- and high-haplotype-diversity genes

To evaluate genes with unusually low or high haplotype diversity independent of gene length, we divided each gene’s median haplotype count by length, applied a log_2_ transform, and standardized values to *z*-scores (*z*) within each level. The analysis highlighted genes at the lower and upper tails of the length-normalized distribution. Among autosomal genes, the lowest-diversity gene in each level was *CHD2* at A (*z* = −4.2), *ATP6V1A* at C (*z *= −4.2), and *RBM5* at T (*z* = −3.8). Conversely, the highest-diversity genes were *HRCT1* at A (*z* = 5.4), *SHISAL1* at C (*z* = 6.7), *CCDC9* at T (*z* = 5.9), and the olfactory receptor *OR4K2* at G (*z* = 5.8). (Supplementary Fig. S2; Supplementary Table S12).

### Haplotype coverage and completeness

As the analysis above indicates, haplotype diversity varies by gene and across A, C, T, and G levels. We therefore quantified JoGo 1.0 performance using two complementary metrics on the 18 095 genes with ≥200 available haploids: (i) cover ratio, the Good-Turing coverage (the probability that a newly sampled haplotype would match one already registered in JoGo 1.0); and (ii) completeness ratio, the Chao1 completeness (the fraction of distinct haplotypes already catalogued out of the predicted total haplotype) [41] (Supplementary Note; Supplementary Tables S13).

Using a pre-specified ≥90% cover ratio as a high-coverage criterion, the fractions of genes meeting this threshold were A (99.1%), C (97.3%), T (91.3%), and G (22.2%) (Supplementary Table S14; Supplementary Fig. S5). In contrast, the completeness ratio shows that a majority of genes reach ≥50% completeness at A (74.2%), C (64.0%), and T (66.4%) but not at G (16.1%) of genes qualified (Supplementary Tables S15; Supplementary Fig. S6). Together, these results indicate that JoGo 1.0 already covers most of the probability mass of common haplotypes at A, C, and T levels (high cover ratio), while a non-trivial tail of rare, distinct haplotypes remains to be discovered—most prominently at the G level (lower completeness).

### ACTG-haplotype IDs across human reference assemblies: GRCh38 and CHM13v2

JoGo 1.0 assigns A-, C-, T-, and G-level haplotype IDs once, by ranking every distinct sequence observed in a combined panel of 258 long-read genomes plus the GRCh38 and CHM13v2 reference assemblies. After this global, frequency-based numbering step, the ranked IDs are mapped separately onto each coordinate system, enabling direct gene-by-gene comparison of globally ranked haplotypes between GRCh38 and CHM13v2. For example, if a gene carries a1 on GRCh38 but a5 on CHM13v2, the single-axis histograms record one occurrence of rank 1 for GRCh38 and one of rank 5 for CHM13v2, and the bubble grid plots a point at (1, 5). Repeating this process for every gene produces a one-dimensional frequency distribution of ACTG-haplotype IDs for each reference and a two-dimensional grid summarizing their co-occurrence (Fig. 2C).

The histograms for the A-level show that haplotypes are heavily concentrated at low ID ranks on both reference assemblies, with frequencies declining steeply as the rank increases. The same pattern is observed for the C, T, and G levels, confirming that the single global ranking is preserved regardless of reference assembly. The accompanying bubble grid shows substantial scatter around the diagonal, indicating that the underlying haplotype sequences often differ between GRCh38 and CHM13v2, and that shared IDs are primarily confined to globally common haplotypes.

As examples of major–minor haplotype pairs between the two assemblies, *EMILIN3* carries a major haplotype (a1) on GRCh38 that is minor on CHM13v2 (a10) (Supplementary Fig. S7), whereas *ADAMTS19* carries a minor haplotype on GRCh38 (a10) that is major on CHM13v2 (a1) (Supplementary Fig. S8). These cases illustrate that either reference alone can miss rare yet potentially functional alleles. By retaining the global frequency landscape while exposing reference-specific biases, JoGo enables users—through its web portal—to instantly determine whether the haplotype encoded in their chosen reference genome is globally common or rare, thereby supporting more accurate variant interpretation, assay design, and population genetic modeling.

### JoGo web server implementation

The JoGo web server is implemented using the Laravel PHP web application framework (https://laravel.com; version 11.11.0) and SQLite3 (https://sqlite.org; version 3.37.2) [42] as the underlying relational database engine. ACTG-haplotype data and associated annotation are stored locally in SQLite databases in read-only mode, with mature indexes to ensure fast query responses and efficient filtering.

The front end employs modern web technologies, including jQuery (https://jquery.com), jQuery UI (https://jqueryui.com), Bootstrap (https://getbootstrap.com), and Font Awesome (https://fontawesome.com) for styling, responsive design, and interactive components. The core UI is rendered using the Roboto font (https://fonts.google.com/specimen/Roboto) for visual consistency. JavaScript modules and CSS resources are compiled and delivered using Laravel Mix, with asset versioning, to facilitate effective cache control.

To support local data analysis and integration of user-provided datasets, JoGo also offers a local haplotype exploration mode. This mode provides downloadable ACTG-haplotype–aligned BAM files that are compatible with the desktop version of the Integrative Genome Viewer (IGV) [43], enabling secure, offline examination of user data alongside JoGo reference haplotypes.

## JoGo web portal functionality

### ACTG-haplotype search

The JoGo web portal provides a flexible and intuitive interface for querying ACTG-haplotype information using a broad range of biological identifiers. From the top page (Fig. 2D), users can search by gene names (e.g. ALDH2), RefSeq transcript and protein identifiers (e.g. NM_000690, NP_000681), Ensembl gene, transcript, and protein IDs (e.g. ENSG00000111275, ENST00000261733, ENSP00000261733), and common variant identifiers such as dbSNP rsIDs (e.g. rs671). JoGo-specific region-based identifiers (e.g. ALDH2_chr12_111761933_111822532) and hierarchical ACTG-haplotype notations—representing increasingly fine-grained levels of sequence resolution—are also supported. Examples include A-level (e.g. ALDH2:a1), C-level (e.g. ALDH2:a1c1), T-level (e.g. ALDH2:a1c1t1), and G-level (e.g. ALDH2:a1c1t1g1).

RefSeq-based haplotype queries that combine transcript IDs and haplotype IDs (e.g. NM_000690:a1) allow seamless navigation from transcript-centric contexts to the corresponding JoGo haplotype records. This flexibility lets researchers from diverse fields, such as clinical genomics, transcriptomics, or population genetics, retrieve ACTG-haplotype data using familiar identifiers.

The search engine automatically maps each query to the corresponding genomic region and returns relevant haplotype structures, population frequencies, and integrated annotations. This design ensures broad accessibility, lowering the barrier for exploring high-resolution haplotype diversity from multiple biological entry points. External resources can link to JoGo using simple URL templates, e.g. https://jogo.csml.org/gene?genename=<gene symbol> for a gene name or https://jogo.csml.org/genicregion?genicregion=<genic region ID> for a genic region. Adding the parameter &format=json to either endpoint (e.g. genename=ALDH2&format=json) returns the same record in machine-readable JSON format, enabling programmatic access and integration into third-party pipelines (full external resource links are provided in Supplementary Table S16).

### ACTG-haplotype search result—*HBB* use case

**Figure 3.**
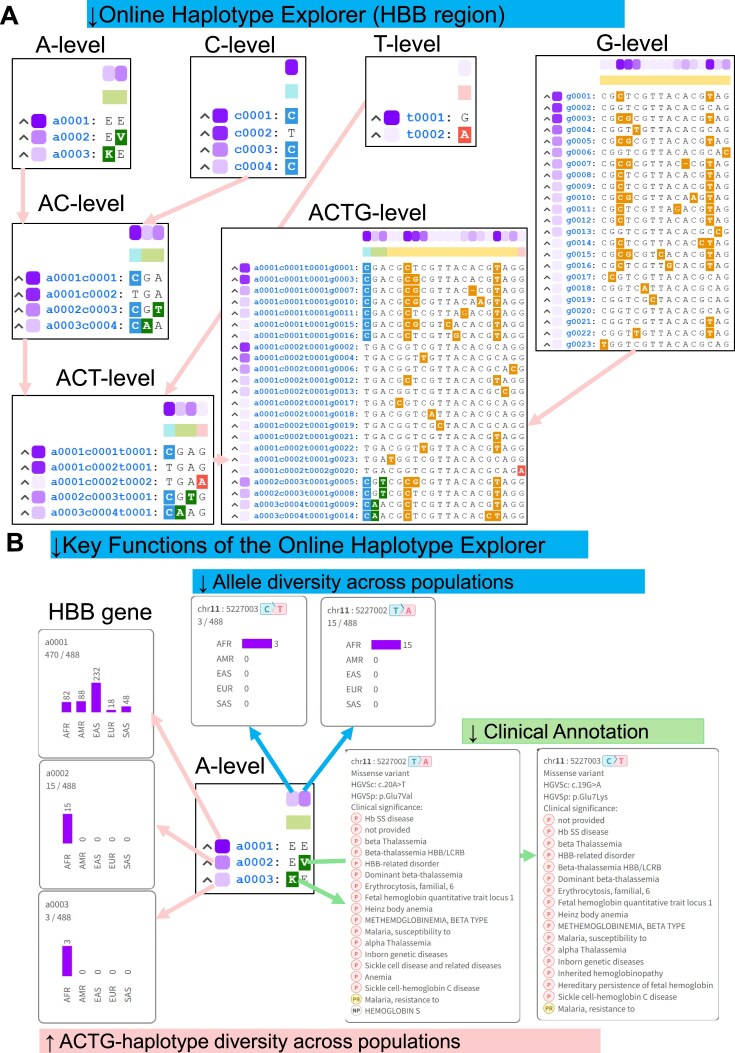
(**A**) Online Haplotype Explorer view for the *HBB* locus, illustrating hierarchical ACTG-haplotype notation from the A-level through the C-level, T-level, and G-level, as well as combined AC-, ACT-, and full ACTG-level haplotype structures. Insets show ranked haplotype IDs (e.g. a1, c1, t1, g1) with representative sequence motifs and color-coded allele differences. (**B**) Detailed view of the Online Haplotype Explorer for A-level haplotypes at the *HBB* locus. The top color bar encodes the global frequency of each variant (darker shading indicates higher frequency), with hover-activated bar-plot tooltips displaying allele counts across JoGo reference populations. The left color bar encodes the global frequency of each A-level haplotype, with similar tooltips for haplotype counts. Clicking or hovering on a variant also reveals ClinVar annotations and provides a direct link to the corresponding record in TogoVar, a companion database to JoGo that aggregates variant annotations.

**Figure 4.**
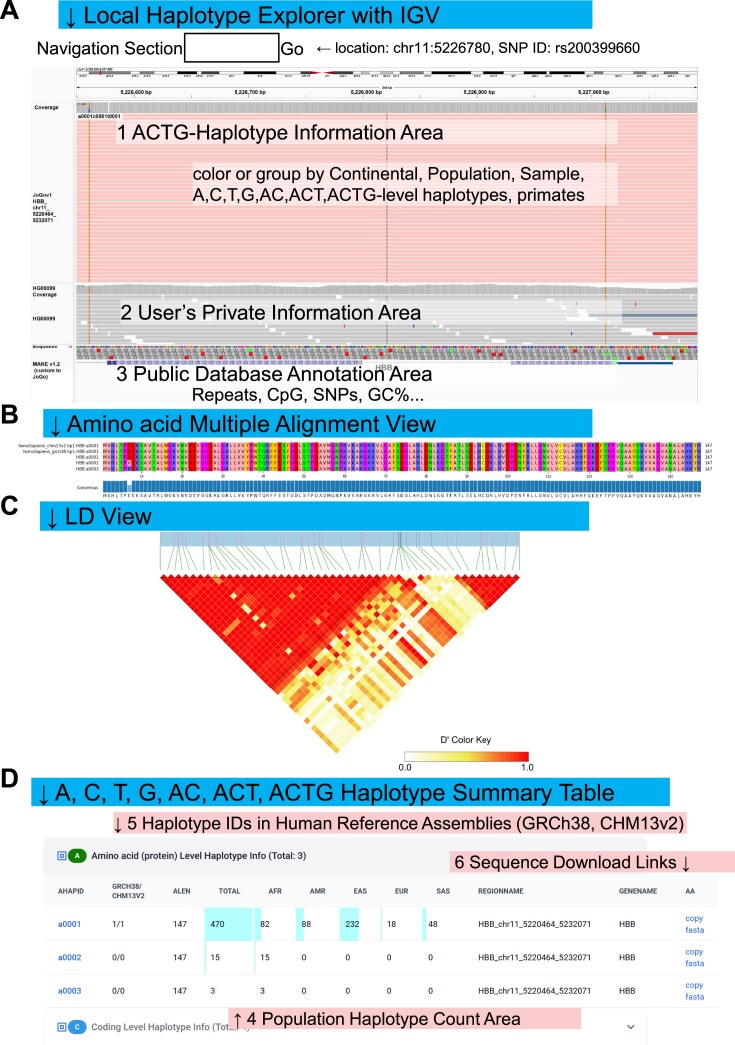
(**A**) Local Haplotype Explorer session for the *HBB* locus in IGV. JoGo’s per-gene ACTG-haplotype dictionary is provided as a pre-aligned BAM, with each “read” representing a haplotype and custom tags for A, C, T, and G (and AC, ACT, and ACTG) IDs, as well as population labels. Public JoGo reference tracks (region 1) and the same public JoGo data loaded privately into IGV (region 2) are displayed together on the GRCh38 coordinate (chr11:5 226 550–5 227 092), enabling secure, side-by-side exploration of shared versus divergent sequence tracts. (**B**) Multiple-sequence alignment of deduced A-level protein haplotypes for *HBB*, including the GRCh38 and CHM13v2 reference coding sequences. (**C**) ACTG-level linkage-disequilibrium (LD) heatmap for the HBB locus. Cells are shaded by pairwise D′ values between coding variants (dark = strong LD; light = weak LD), indicating haplotype structure at the nucleotide level. (**D**) Population-specific counts of A-level HBB haplotypes across the five JoGo reference populations (EAS, AFR, AMR, SAS, EUR). Bars indicate the number of distinct A-level haplotypes observed in each population, allowing for a comparison of haplotype diversity within and between populations.

When a user enters a gene symbol or other identifier in the search box (Fig. 2D), such as *HBB*, the portal launches an integrated gene view that consolidates all four ACTG-haplotype layers for that locus. Two complementary visual tools form the core of this view: the Online Haplotype Explorer (Fig. 3; described in a later section), which highlights amino acid and nucleotide differences among haplotypes, and the Local Haplotype Explorer (Fig. 4; described later), a complete base-resolution workspace that can also incorporate user-supplied, ethically protected datasets for side-by-side analysis with the public JoGo reference panel. Surrounding these viewers, JoGo provides a multiple-alignment viewer of deduced A-level protein sequences (Fig. 4B), an ACTG-level linkage-disequilibrium heatmap (Fig. 4C), population-diversity summaries for every hierarchical level (Fig. 4D), and a catalogue-assignment matrix indicating which reference individuals carry each haplotype. A variant-to-haplotype table lists all distinguishing SNPs and indels, along with online or local links that either highlight the variant in the local viewer or center the online viewer on its genomic position. Finally, outbound links connect the page to (i) genome cohort databases such as PheWeb instances for UK Biobank and TOPMed [44], FinnGen [45], PhewebJP [46], and KoGES [47]; (ii) model organism phenotype databases for mouse [48] and zebrafish [49]; (iii) clinical-interpretation resources including ClinVar [22], ClinGen [50], the NIH Genetic Testing Registry [51], DECIPHER [52], and ClinVar Miner [53]; and (iv) general annotation resources including GeneCards [54], Open Targets [55], Human Protein Atlas [56], gnomAD [57], and TogoVar [58]. These links enable users to move seamlessly from JoGo’s high-resolution haplotype environment to broader public functional annotations.

### Usage of online haplotype explorer—*HBB* use case

The Online Haplotype Explorer provides an in-browser canvas for rapid, interactive comparison of ACTG-haplotypes. Figure 3 demonstrates its functionality for the *HBB* locus. Users can toggle among the A, C, T, G, AC, ACT, and ACTG levels to reveal progressively finer sequence differences (Fig. 3A).

For each haplotype, a colored rectangle along the top margin encodes its global variant frequency (darker shades indicate higher frequencies). In contrast, a corresponding rectangle along the left margin encodes the haplotype’s overall frequency. Hovering over either rectangle triggers a bar-plot tooltip displaying counts across all JoGo reference populations. Variant positions within the alignment are similarly marked by shaded rectangles whose intensity reflects their global frequency, with hover tooltips revealing population-specific counts (top and left bar plots in Fig. 3B).

Hovering over a variant also opens an annotation panel that, when ClinVar [22] and MGeND [59] entries are available, lists associated phenotypes and clinical significance, linking the haplotype structure directly to medical context (right-hand tooltip in Fig. 3B). Clicking the variant opens its detailed record in TogoVar, JoGo’s companion database, where users can inspect aggregated variant annotations [58]. From TogoVar, a direct link leads to the ClinVar Variation (VCV) page (Supplementary Fig. S9A and B), from which users can navigate to condition-specific ClinVar record (RCV) pages that provide clinical assertions and supporting evidence (Supplementary Fig. S9C–E). In this way, the Online Haplotype Explorer enables seamless drill-down from haplotype-level context in JoGo 1.0, through TogoVar, to variant and clinical evidence in ClinVar, thereby making the provenance of each clinical assertion transparent and facilitating user-led evaluation and follow-up experimental design (Supplementary Fig. S9).

### Usage of local haplotype explorer—*HBB* use case

The Local Haplotype Explorer (Fig. 4) enables advanced, privacy-preserving analysis by loading JoGo’s per-gene ACTG-haplotype dictionaries directly into a locally installed instance of the IGV [43]. Because all operations are executed on the user’s local computing environment, sensitive human genome datasets (Fig. 4A, region 2) can be integrated securely with the public JoGo reference panel (Fig. 4A, region 1).

Whereas the Online Explorer emphasizes pairwise differences among haplotypes, the Local Explorer also visualizes sequence tracts shared across haplotypes and maps every allele onto the familiar GRCh38 human reference assembly coordinate system. Each dictionary is provided as a pre-aligned BAM file in which each “read” corresponds to a single haplotype, with custom tags encoding A-, C-, T-, G-, AC-, ACT-, and ACTG-level IDs along with population labels, enabling color-coding or grouping by haplotype level, population, or sample. Figure 4A shows an IGV session for *HBB* (zoomed to chr11:5 226 550–5 227 092).

In the public database annotation area, users can load track-based resources, including GC% content, public SNP databases, and repeat regions. Additionally, JoGo supplies Iso-Seq sequence alignments for 21 tissues, including 164 publicly available long-read Iso-Seq datasets (Supplementary Fig. S10 and Supplementary Table S17), which enables the inspection of haplotype-specific transcript structures and isoform usage within the same genomic window. Together, these resources allow researchers to compare ACTG-haplotypes with the reference genome, integrate them with private data, and explore allele-specific expression or splicing patterns with the complete flexibility of a desktop IGV environment.

### ACTG-haplotype QTL

JoGo provides direct interrogation of the relationship between ACTG-haplotype structure and gene expression, extending beyond conventional eQTL studies that focus on single variants. In JoGo 1.0, we analyzed 1280 samples passing QC from three independent LCL RNA-seq panels: (i) 462 HapMap European and African samples [25], (ii) an expanded panel of 731 HapMap samples selected to balance ancestry representation [26], and (iii) 87 HapMap Japanese samples analyzed under interferon stimulation [27]. For each dataset, JoGo performs pairwise haplotype–expression association testing (“haplotype QTL”) at the A, C, T, AC, and ACT levels.

**Figure 5.**
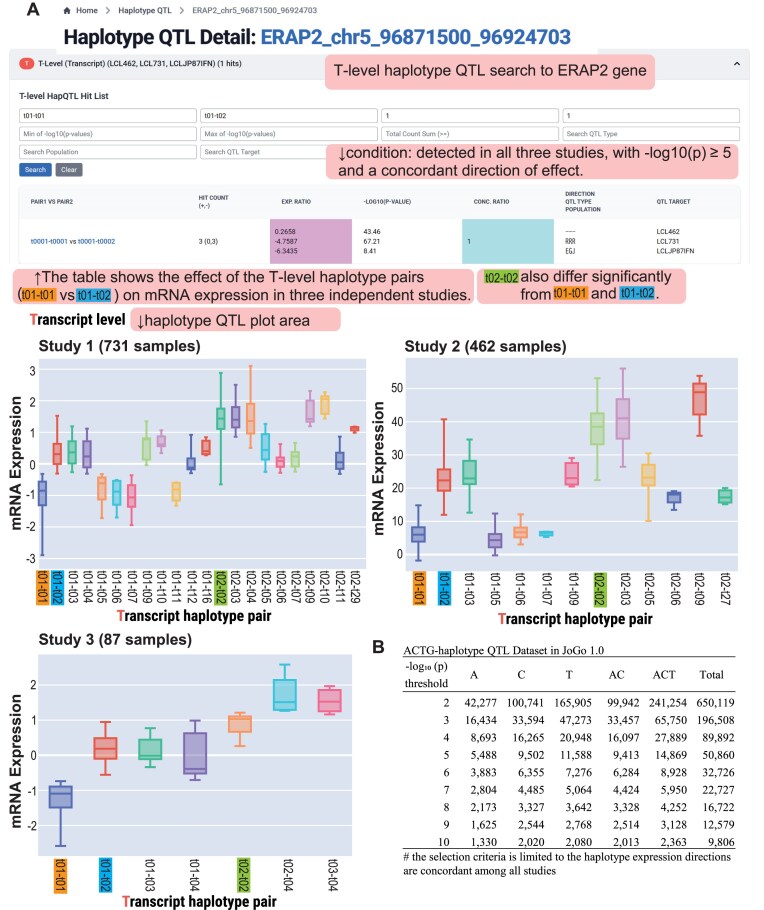
(**A**) Haplotype QTL detail view for the ERAP2 locus. The top toolbar allows users to select the haplotype level (A, C, or T), apply significance filters (–log_10_(*p*) ≥ 5), and apply concordance filters (the same effect direction across all three studies). The middle section displays a table for the selected T-level comparison (t1–t1 versus t1–t2), reporting the number of significant hits, fold change, –log_10_(*p*), and concordance ratio. The bottom panel shows boxplots of normalized messenger RNA (mRNA) expression stratified by haplotype pair for Study 1 (*n* = 731), Study 2 (*n* = 462), and Study 3 (*n* = 87), illustrating the reproducibility of the QTL effect. The t2–t2 group exhibits higher mRNA expression than the t1–t1 and t1–t2 groups across all three studies. (**B**) Summary table of significant haplotype QTL associations in JoGo 1.0. For each ACTG level (A, C, T, AC, and ACT), counts of associations are shown at increasing –log_10_(*p*) thresholds (2 through 10), restricted to associations with concordant effect direction in all three datasets.

By incorporating complete gene-level haplotype architecture, the haplotype QTL framework evaluates the combined impact of multiple linked variants within a single, biologically coherent unit. Across the three datasets, 650 119 significant haplotype QTL associations with concordant effect directions passed the pairwise comparison filter (summary counts for each ACTG level and p-value threshold are shown in Fig. 5B).

The haplotype QTL database is accessible through the JoGo portal’s haplotype QTL section (Fig. 5A, top). For example, querying the *ERAP2* gene and selecting a haplotype pair with a concordance ratio of 1 and significant *p*-values across all three studies returns a results table (Fig. 5A, middle) summarizing statistics for each haplotype pair (e.g. t1–t1 versus t1–t2). The associated plot area (Fig. 5A, bottom) displays boxplots of normalized mRNA expression by haplotype pair for Studies 1 (*n* = 731), 2 (*n* = 462), and 3 (*n* = 87), illustrating the reproducibility of the QTL effect. The t2–t2 group exhibits higher median mRNA expression than the t1–t1 and t1–t2 groups across all three studies.

Every significant haplotype QTL entry is deep-linked to its exact ACTG sequence, enabling users to move directly from an expression contrast (e.g. t1–t1 < t1–t2 < t2–t2 in *ERAP2*) to the underlying 3′ and 5′ UTR variant constellation. This integration provides rapid, nucleotide-resolved insight into how haplotype architectures may shape gene regulation and phenotype or disease risk. Crucially, by coupling the hierarchical haplotype levels with the exact ACTG constellations behind each association, JoGo serves as an actionable portal—helping users prioritize targets and generate hypotheses for experimental validation (e.g. UTR-focused luciferase reporters, minigene constructs, and isogenic CRISPR edits) to establish functional impact. The interface also aggregates expression metrics across all observed haplotypes—including rare ones—allowing users to interpret standard signals in the context of population diversity and identify outlier haplotypes with potentially unique regulatory effects.

## Discussion

JoGo 1.0 is the first genome-wide, long-read–based reference of human gene haplotypes, integrating uniform ACTG identifiers with an open web portal for exploration and analysis. It covers 19 194 MANE-standardized genes and corresponding transcripts. From 258 high-quality HiFi genomes, including 108 generated in-house, we cataloged 174 376 A-level (amino acid), 300 610 C-level (coding sequence), 486 288 T-level (transcript), and 3 695 204 G-level (gene) haplotypes, assigning uniform identifiers through the novel ACTG hierarchical nomenclature. The publicly accessible web portal supports mapping from variants, genes, and haplotype IDs to ACTG-haplotype information, integrating variant annotation with association data from 1280 HapMap transcriptomes across three studies to enable single-haplotype-level QTL analysis. For privacy-sensitive projects, the Local Haplotype Explorer provides base-resolution ACTG-haplotype information directly to a user’s local computing environment, enabling the secure integration of confidential datasets without requiring cloud upload.

## Perspective

### Increase sample size and cross-version consistency

We will substantially expand the long-read cohort beyond JoGo 1.0 (258 genomes across 19 194 MANE genes), prioritizing underrepresented groups (e.g. EUR, AFR subpopulations, and Indigenous cohorts). This will increase both sample size and the number of catalogued haplotypes, yielding more representative global frequency estimates. With each major release (e.g. JoGo 2.0), JoGo reassigns ACTG identifiers strictly by descending global frequency on the expanded cohort (no ID locking across versions). The primary information in JoGo is the sequence–ID pair, and all interpretation is anchored to this mapping. To ensure cross-release consistency, we will implement a dedicated haplotype liftover framework—conceptually analogous to coordinate liftover between GRCh38 and CHM13v2, but operating at the ACTG-haplotype level. This will provide an exact cross-version sequence–ID mapping (e.g. between JoGo 1.0 and 2.0) whenever the identical sequence is observed; newly observed or discontinued sequences will naturally have no mapping. As cohort diversity increases, rank shifts are expected primarily for rare or population-specific haplotypes; given the current underrepresentation of some groups (e.g. EUR) in JoGo 1.0, shifts affecting haplotypes currently labeled as major are expected to be exceptionally rare, yet cannot be entirely excluded. By combining (i) cohort expansion and (ii) sequence-anchored cross-version mapping, future JoGo releases will preserve interpretability while allowing frequency ranks to reflect more representative global data.

### Tissue-aware haplotype-QTL and multi-omics integration

JoGo 1.0 already links GTEx eQTL variants to ACTG haplotypes, enabling variant-to-haplotype exploration and interpretation; however, direct, tissue-resolved associations between ACTG haplotypes and gene expression are currently available only for LCLs. As proof of concept, JoGo 1.0 includes haplotype-QTL results in three independent LCL cohorts (*n *= 731, 462, 87; Fig. 5), which serve as the methodological anchor for generalizing to a multi-tissue setting. Building on this framework, future JoGo releases will compute direct, haplotype-level QTLs across multiple tissues (e.g. using GTEx) and progressively integrate multi-omics layers (e.g. proteome, metabolome), mapping all readouts to ACTG haplotypes within the same ACTG-haplotype-anchored framework.

### Evolutionary depth

JoGo 1.0 already enables base-resolution inspection of human ACTG haplotypes alongside primate reference sequences in the Local Haplotype Explorer (Fig. 4). Building on this, future releases will display, for each human haplotype, a percent-identity conservation score (how similar the human haplotype is to the orthologous primate sequence) to each of four ape references (chimpanzee, bonobo, gorilla, and orangutan), computed within the transcript-anchored syntenic window (AC-, ACT, and ACTG-level), on the JoGo portal (e.g. alignment-based similarity heat maps visualizing pairwise percent identity between human ACTG-haplotypes and primate haplotype reference sequences).

### From each gene-level unit to the structure of gene-level units

In JoGo 1.0, we concentrated on per-gene haplotype diversity using the ACTG-haplotype notation (ACTG-gene unit). Future releases will integrate the latest T2T assemblies from pangenome projects (HPRC [32], Human Pangenome Project [60], Chinese pangenome [61], JaSaPaGe [31], and others) as the primary coordinate backbone to represent the order and orientation of ACTG-gene units along contiguous sequence segments. We term this segment-level representation the list of ACTG-gene units (LAG). The notation already supports isoform-specific haplotypes for every transcript and will be extended to non-coding RNAs (T- and G-levels) and intergenic regulatory regions (R-level), enabling richer segment-level LAGs. For coordinate projection to T2T, we will employ liftOn-based approaches [62] anchored on GFF-based gene models (e.g. MANE annotation). We view T2T as complementary to JoGo 1.0—ACTG-IDs define the units, and T2T assemblies define how those units are arranged—thereby moving from per-gene catalogs to ordered and multi-gene contexts. Complex loci with copy-number variation, such as *LILRB3*–*LILRA6* [63], will be represented by per-copy ACTG IDs.

### Other perspectives

We are also developing tools to infer ACTG-haplotype pairs from short-read sequencing or genotyping arrays—analogous to HIBAG [64], HLA-VBSeq [65], or T1K [66]—and plan to integrate eQTL, GWAS, and other haplotype-centric association results into the portal.

### Limitations and mitigation

Because JoGo 1.0 is based on non-T2T references, residual phasing (switch) errors may persist. Frequency-based IDs reflect only the current 258 genomes and may reorder for less frequent haplotypes as sample diversity increases. These issues will be addressed through the incorporation of higher-quality T2T assemblies and the expansion of cohorts in future releases. Through these technical and methodological advances, JoGo aims to serve as a resource for dissecting human haplotype diversity, accelerating precision medicine research, and enabling the discovery of novel biomarkers and therapeutic targets.

## Supplementary Material

gkaf1232_Supplemental_Files

## Data Availability

The JoGo project is carried out under the auspices of the Japan Science and Technology Agency (JST) Database Integration Coordination Program (DICP). The current public release, JoGo 1.0, is available at https://jogo.csml.org with permanent, free access (no login required). All ACTG-haplotype identifiers, corresponding sequences, and population frequency tables are retrievable via the portal and released under the Open Database License (ODbL 1.0); portal text and figures are available under CC BY-SA 4.0. Raw PacBio HiFi FASTQ files for the 108 HapMap samples generated in this study are deposited at the DNA Data Bank of Japan under DDBJ BioProject (PRJDB18487). Third-party annotations (e.g. GTEx, ClinVar, NHGRI-EBI GWAS Catalog) are subject to their respective licenses.

## Appendix: Author list of the members in the Variant Information Standardization Collegium (VISC)

All members contributed to the discussions on the JoGo haplotype database and its implementation at VISC or related internal meetings.

Methodology Supporting: Contributed to the discussion and refinement of implementation strategies.

Masao Nagasaki^1,2^

Toshiaki Katayama^3^

Yuki Moriya^3^

Shuichi Kawashima^3^

Shuto Machida^1^

Taichi Matsubara^1^

Akio Nagano^4^

Riu Yamashita^5,6^

Toyoyuki Takada^7^

Nobutaka Mitsuhashi^3^

Takatomo Fujisawa^10^

Mayumi Kamada^8^

Daisuke Satoh^11^

Tsuyoshi Hachiya^12^

Masaki Ohira^13^

Yoko Kuroki^14^

Patricia Severino^1^

Samuel Papa Kwesi Owusu^1^

Yifan Huangfu^1^

Atsushi Doi^1^

Yuichiro Hara^8^

Minae Kawashima^3^

Yosuke Kawai^9,10^

^11^ Kamonohashi Inc., 2–53 Chuo, Moriya, Ibaraki, 302–0115, Japan

^12^ Genome Analytics Japan Inc., Tomihisa-cho, Shinjuku-ku, Tokyo, 162-0067, Japan

^13^ Department of Integrated Biosciences, Graduate School of Frontier Sciences, The University of Tokyo, Chiba, 277-8562, Japan

^14^ Department of Genome Medicine, National Center for Child Health and Development, Tokyo, 157-8535, Japan
