# Supplementary material for: JoGo 1.0: the ACTG hierarchical nomenclature and database covering 4.7 million haplotypes across 19,194 human genes
Source: Nucleic Acids Res. 2025 Nov 29;54(D1):D1159–73. doi: 10.1093/nar/gkaf1232 (PMC12807767; doi:10.1093/nar/gkaf1232)
Supplement: gkaf1232_Supplemental_Files [file gkaf1232_supplemental_files.zip › Supplementary Figures_revision1.pdf]

Supplementary Figures

JoGo 1.0: the ACTG hierarchical nomenclature and database covering 4.7 million haplotypes across 19,194 human genes

Supplementary Figure 1

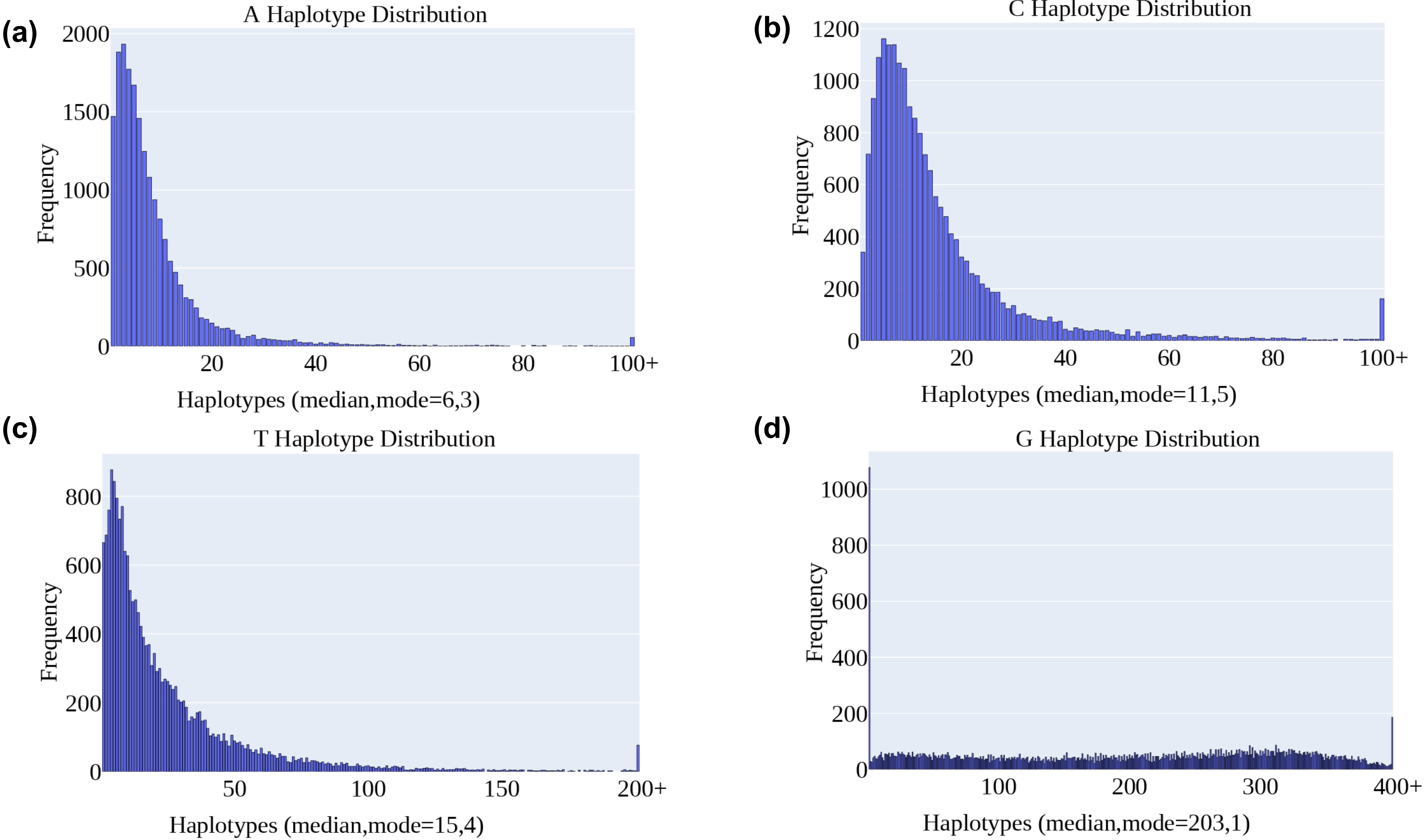

Continued.

Supplementary Figure 1 | Across-gene distributions of haplotype counts at the A, C, T, and G levels in JoGo 1.0.

Histograms show, for each gene (n = 19,194), the number of distinct haplotypes observed at the indicated ACTG level across the cohort of assembled haploid chromosomes. Panels: (a) A-level, (b) C-level, (c) T-level, (d) G-level. The x-axis is the haplotype count per gene; the y-axis is the number of genes (frequency). Numbers in parentheses under each x-axis give the median and mode of the distribution across genes: A (6, 3), C (11, 5), T (15, 4), G (203, 1). Rightmost bins aggregate genes with counts ≥100 (A, C), ≥200 (T), or ≥400 (G).

Supplementary Figure 2

(a)

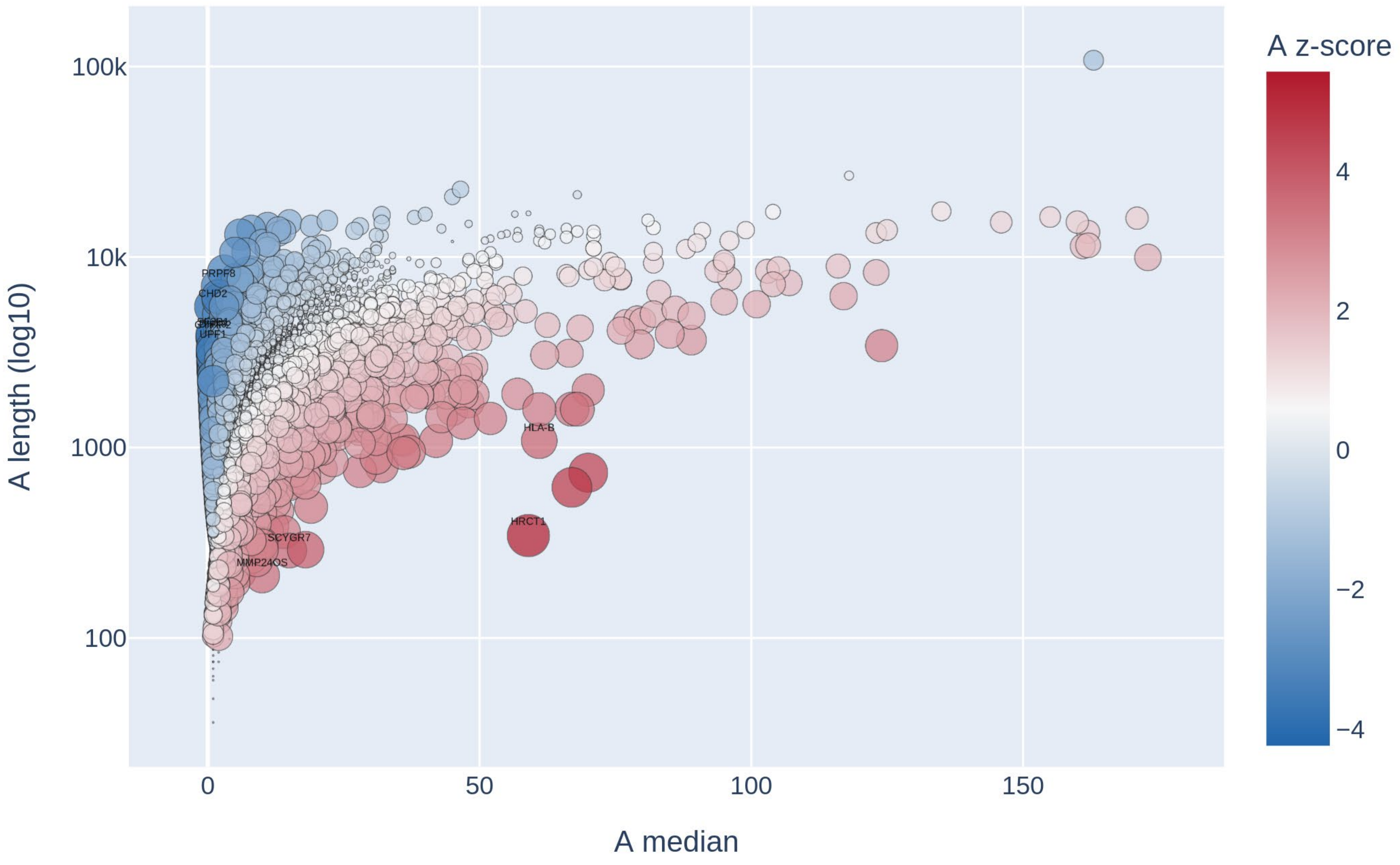

$R^2 = 0.39$ $\beta = 122$ bp per distinct haplotype

Supplementary Figure 2

(b)

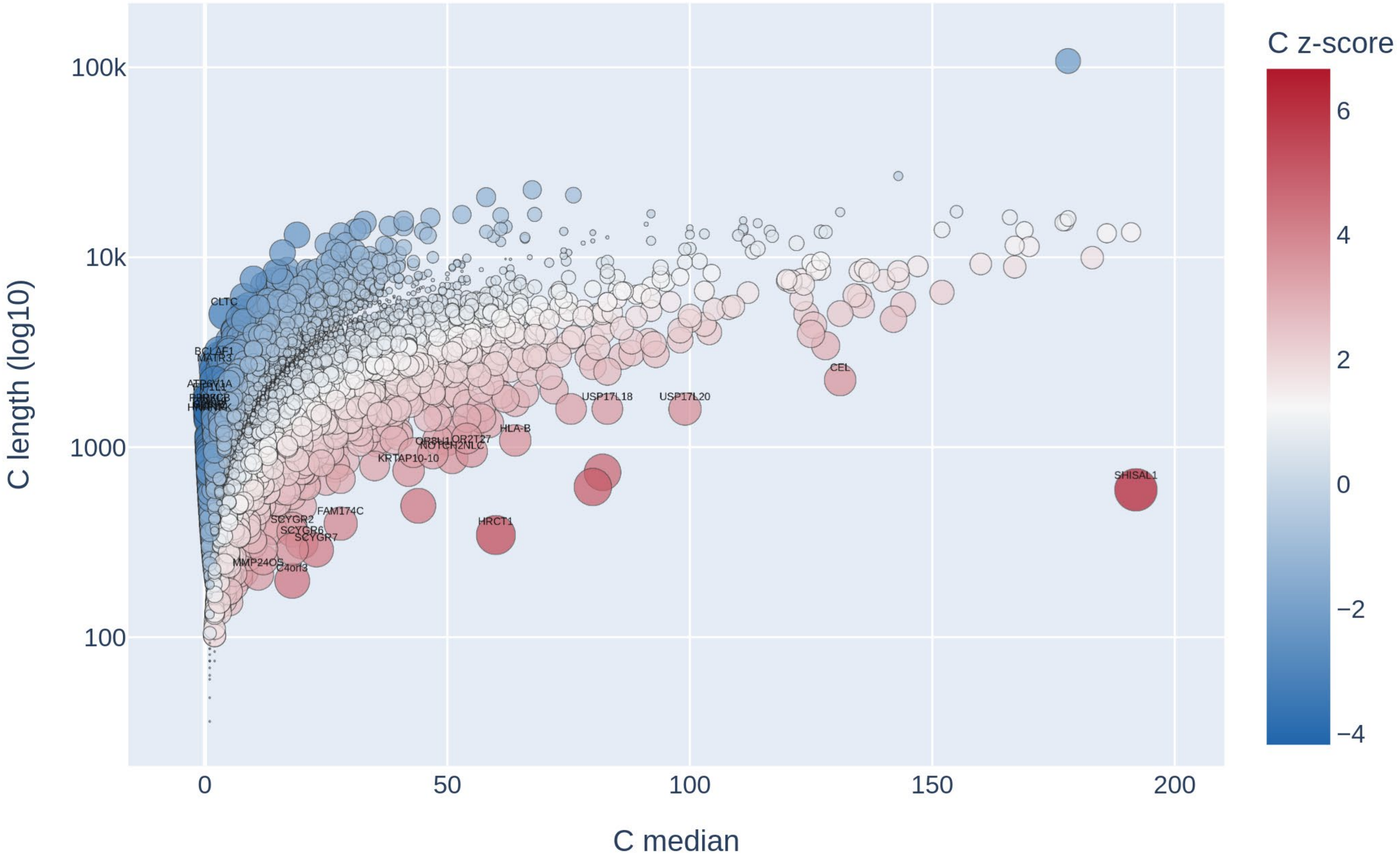

$R^2 = 0.44$ $\beta = 84$ bp per distinct haplotype

Supplementary Figure 2

(c)

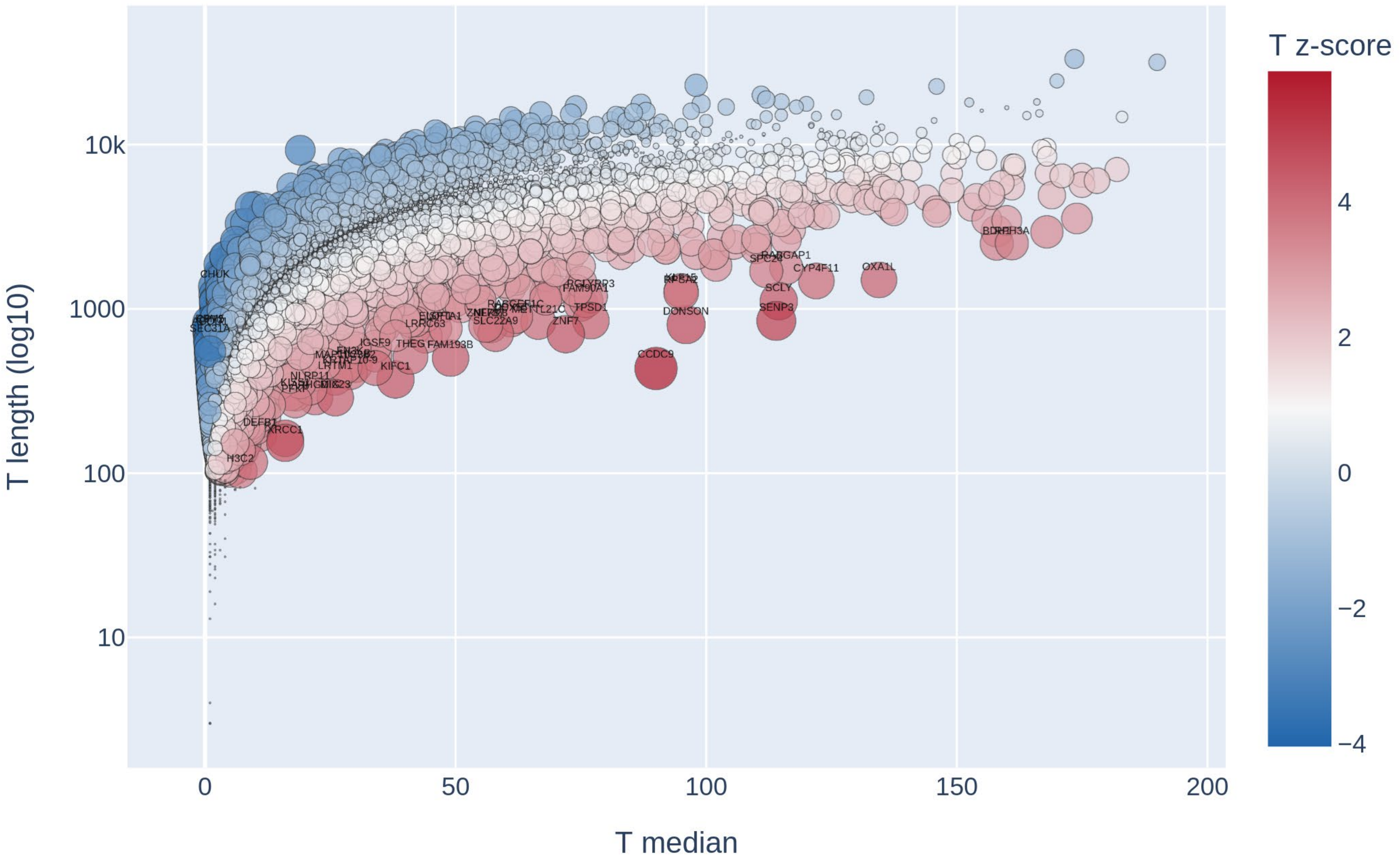

$R^2 = 0.62$ $\beta = 76$ bp per distinct haplotype

Supplementary Figure 2

(d)

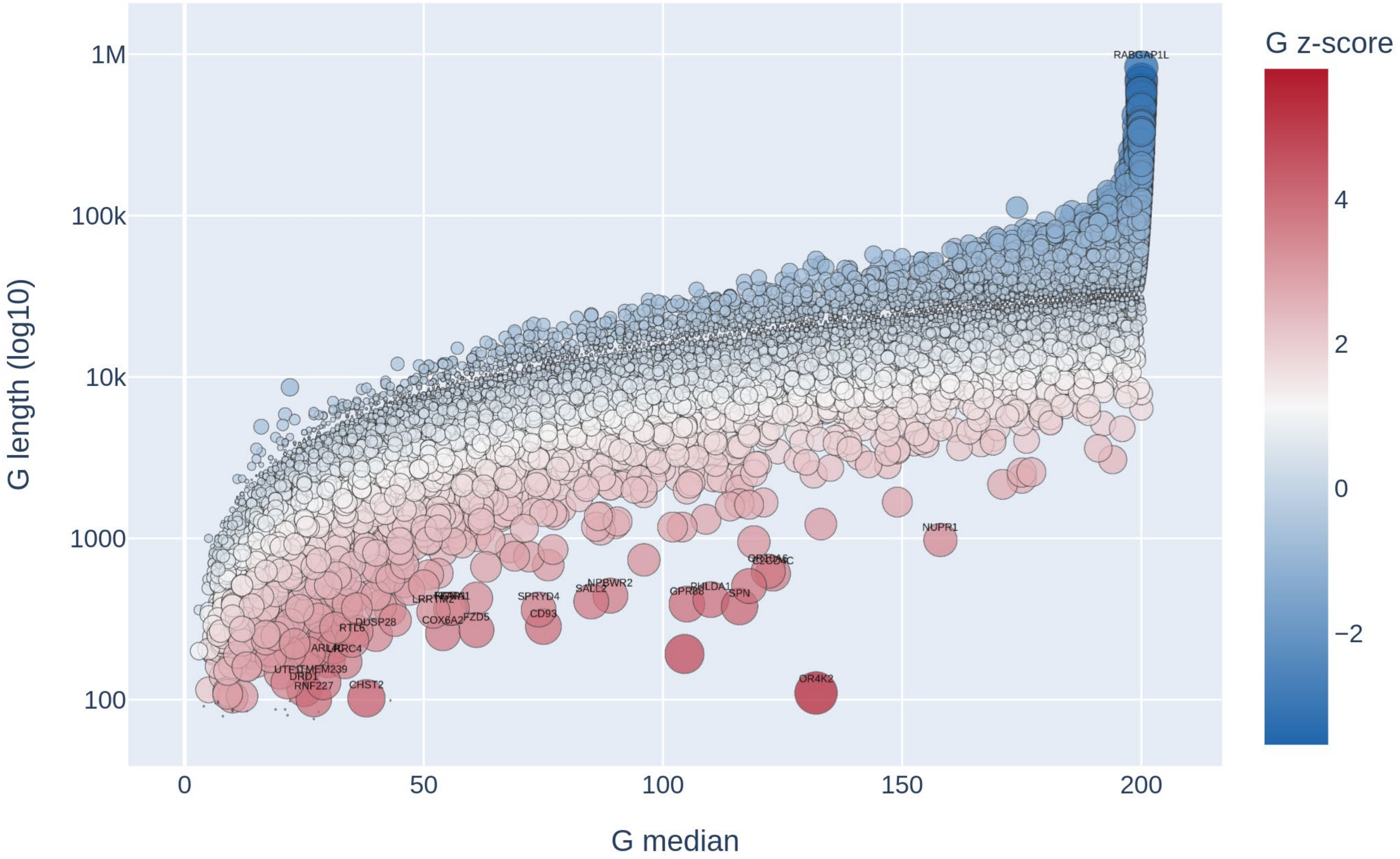

$R^2 = 0.35$ $\beta = 531$ bp per distinct haplotype

Continued.

Supplementary Figure 2 | Relationship between haplotype diversity and sequence-context length across ACTG levels.

Panels show (a) A-level, (b) C-level, (c) T-level, and (d) G-level. Each point is one genic region (n = 18,095 with ≥200 haploids in JoGo 1.0). The x-axis is the median number of distinct haplotypes at the indicated level (a|c|t|g_median) computed from 100 random subsamplings of 200 haploid chromosomes; the y-axis is the corresponding sequence-context length (a|c|t|g_length, bp; log_{10} scale). Point color encodes the standardized log_2 haplotype density per base (a|c|t|g_zscore of log_2(median/length); blue = lower, red = higher). Gene labels are shown only for z-scores ≤ −3.5 or ≥ 3.5. The R^2 of the x–y correlation in each panel is shown below.

Supplementary Figure 3 ACTG-haplotype count view for each genic region.

Joint Open Genome and Omics Platform (JoGo)

Home > ACTG Haplotype Summary by Genic Region

ACTG Haplotype Summary by Genic Region

Search

Region Name	Gene Name	Chromosome	Start
Search region name	Input genename here	Select Chromosome	Start position
**End**	**Show**	**Min A**	**Max A**
End position	50 records	Min A count	Max A count
**Min C**	**Max C**	**Min T**	**Max T**
Min C count	Max C count	Min T count	Max T count
**Min G**	**Max G**	**Sort By**	**Order by**
Min G count	Max G count	Sort by	Ascending

Search

Showing 1 to 50 of 19194 results

< 1 2 3 4 5 6 7 8 9 10 ... 383 384 >

Detail	Region Name	Gene Name	Chr	Start	End	ACTG	ACT	AC	A	C	T	G
Detail	A1BG_chr19_58340183_58358492	A1BG	chr19	58,340,183	58,358,492	101	37	12	10	12	21	101
Detail	A1CF_chr10_50794409_50890627	A1CF	chr10	50,794,409	50,890,627	287	59	12	3	12	49	287
Detail	A2ML1_chr12_8817621_8881787	A2ML1	chr12	8,817,621	8,881,787	361	90	71	41	71	8	361
Detail	A2M_chr12_9062708_9120919	A2M	chr12	9,062,708	9,120,919	278	30	27	16	27	3	278
Detail	A3GALT2_chr1_33301766_33326098	A3GALT2	chr1	33,301,766	33,326,098	259	15	15	12	15	1	259
Detail	A4GALT_chr22_42687121_42725870	A4GALT	chr22	42,687,121	42,725,870	396	38	13	6	13	27	396
Detail	A4GNT_chr3_138118713_138137390	A4GNT	chr3	138,118,713	138,137,390	200	24	12	7	12	12	200

Supplementary Figure 4

The top and bottom genes by haplotype count.

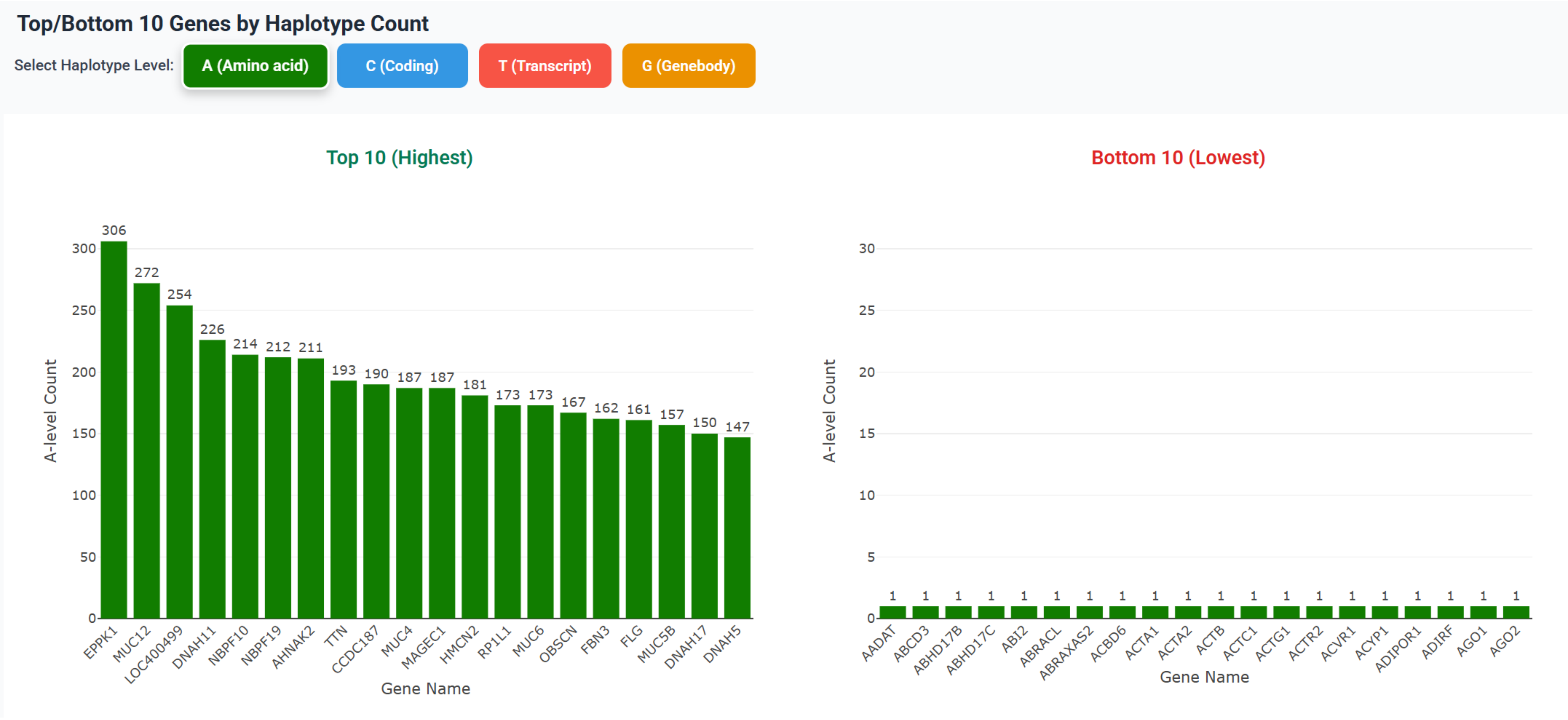

Supplementary Figure 5

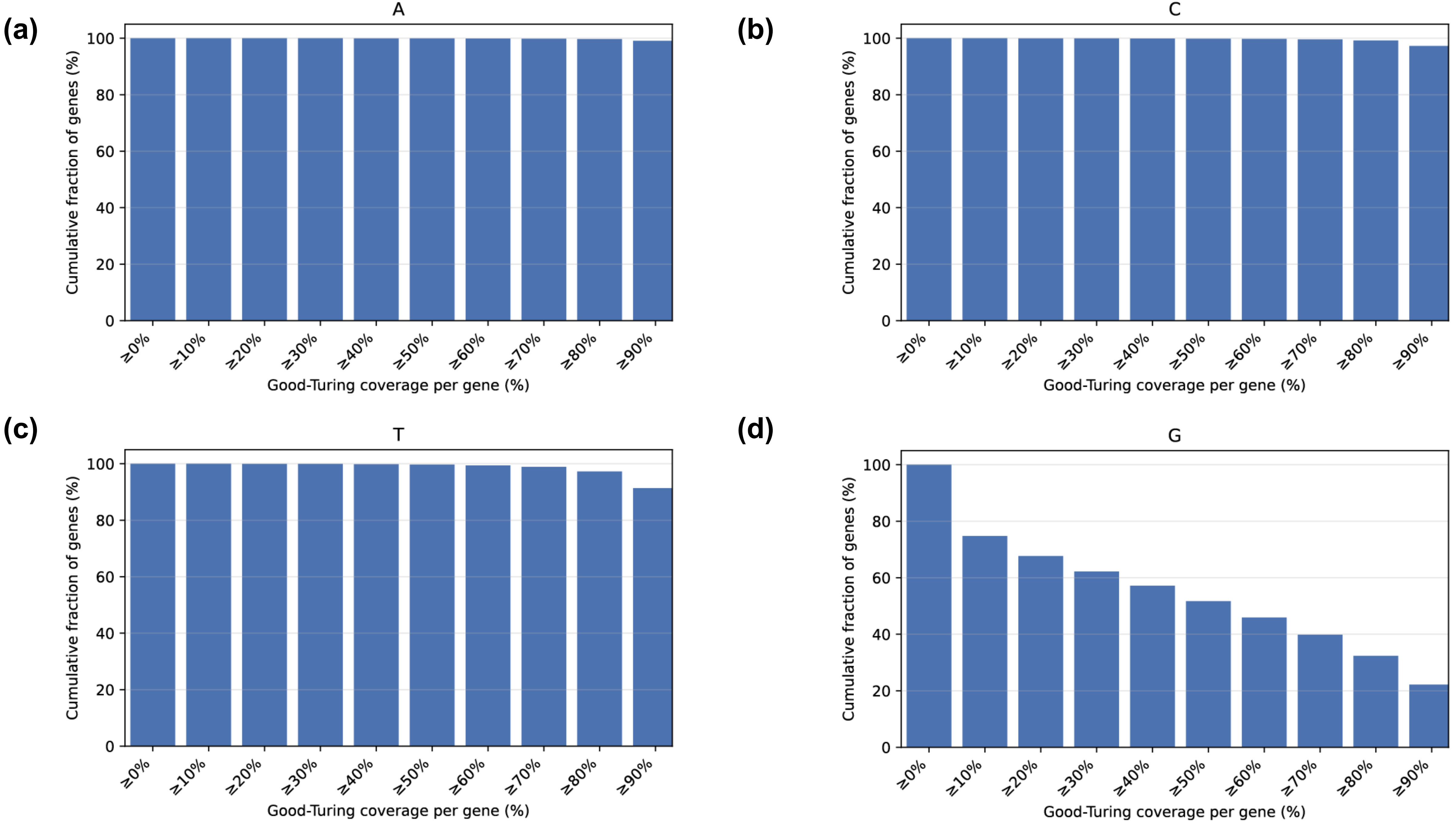

Continued.

Supplementary Figure 5 | Cumulative distribution of Good–Turing coverage of gene-level haplotypes across ACTG levels.

For (a) A-, (b) C-, (c) T-, and (d) G-level haplotypes, the cumulative fraction of genes is plotted against Good–Turing coverage per gene (%), evaluated on JoGo 1.0 and restricted to the 18,095 genes with ≥200 available haploids. Good–Turing coverage (1 − estimated unseen mass) quantifies the proportion of sampling probability already assigned to haplotypes that are observed (already registered haplotypes in JoGo 1.0), in contrast to the Chao1-based completeness metric that estimates the fraction of total richness recovered. The x-axis shows coverage thresholds (≥0%–≥90%); the y-axis shows the cumulative fraction of genes meeting each threshold.

Supplementary Figure 6

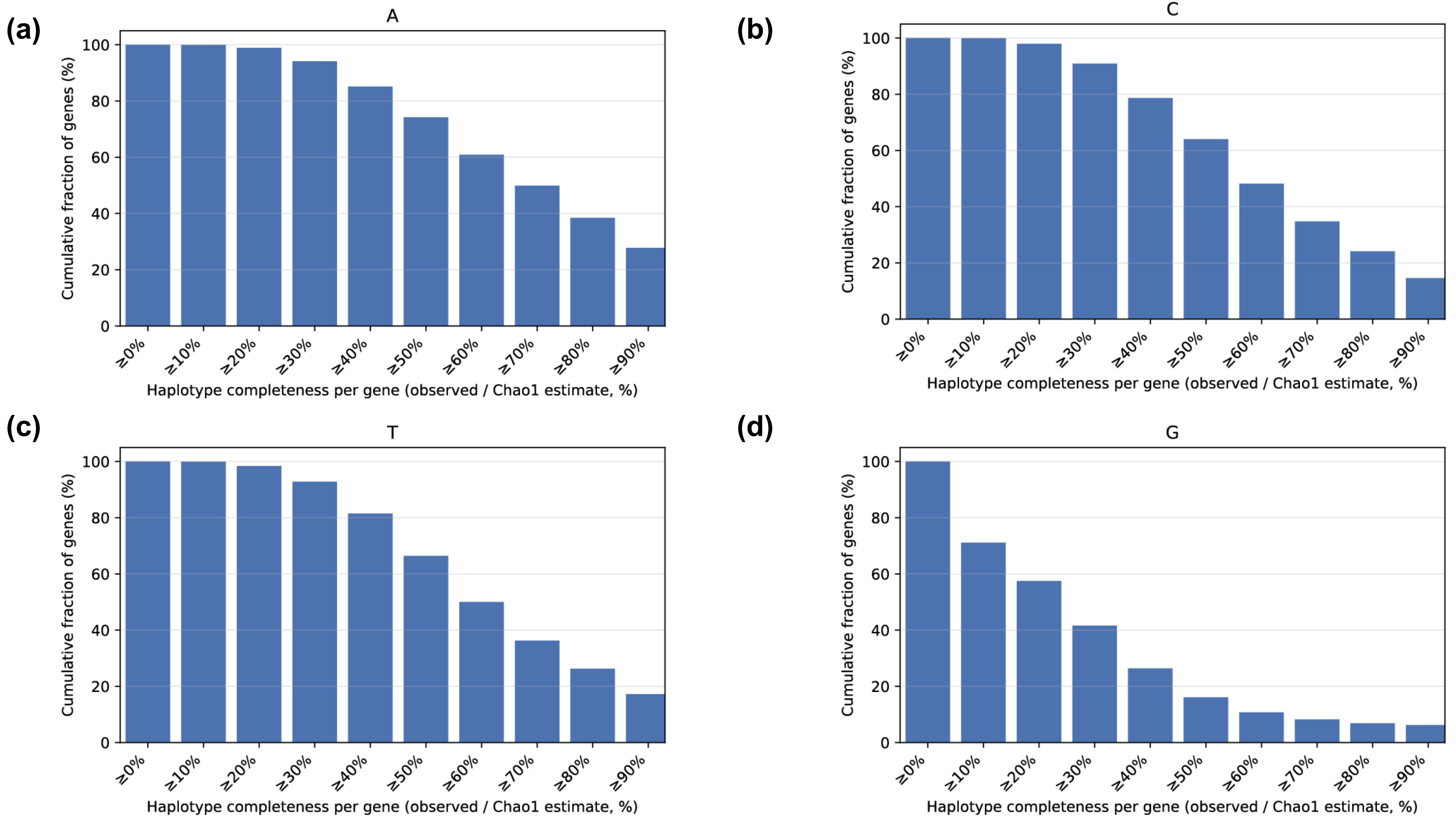

Continued.

Supplementary Figure 6 | Cumulative distribution of gene-level haplotype completeness across ACTG levels.

For (a) A-, (b) C-, (c) T-, and (d) G-level haplotypes, the cumulative fraction of genes is plotted against haplotype completeness per gene (observed/Chao1 estimate, %), using the 18,095 genes with ≥200 available haploids. The x-axis shows completeness thresholds (≥0%–≥90%); the y-axis shows the cumulative fraction of genes meeting each threshold.

Supplementary Figure 7

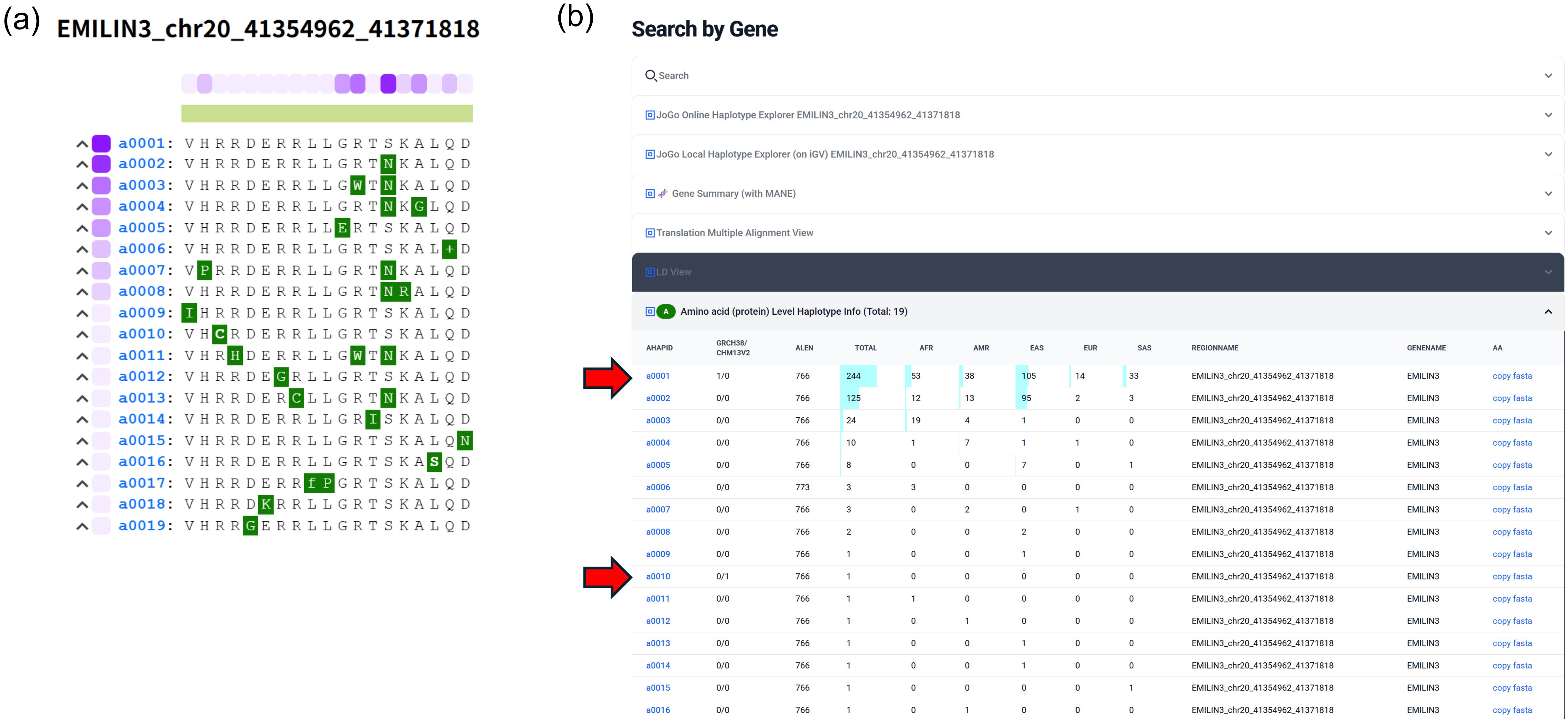

AHAPID	GRCH38/ CHM13V2	ALEN	TOTAL	AFR	AMR	EAS	EUR	SAS	REGIONNAME	GENENAME	AA
a0001	1/0	766	244	53	38	105	14	33	EMILIN3_chr20_41354962_41371818	EMILIN3	copy fasta
a0002	0/0	766	125	12	13	95	2	3	EMILIN3_chr20_41354962_41371818	EMILIN3	copy fasta
a0003	0/0	766	24	19	4	1	0	0	EMILIN3_chr20_41354962_41371818	EMILIN3	copy fasta
a0004	0/0	766	10	1	7	1	1	0	EMILIN3_chr20_41354962_41371818	EMILIN3	copy fasta
a0005	0/0	766	8	0	0	7	0	1	EMILIN3_chr20_41354962_41371818	EMILIN3	copy fasta
a0006	0/0	773	3	3	0	0	0	0	EMILIN3_chr20_41354962_41371818	EMILIN3	copy fasta
a0007	0/0	766	3	0	2	0	1	0	EMILIN3_chr20_41354962_41371818	EMILIN3	copy fasta
a0008	0/0	766	2	0	0	2	0	0	EMILIN3_chr20_41354962_41371818	EMILIN3	copy fasta
a0009	0/0	766	1	0	0	1	0	0	EMILIN3_chr20_41354962_41371818	EMILIN3	copy fasta
a0010	0/1	766	1	0	0	0	0	0	EMILIN3_chr20_41354962_41371818	EMILIN3	copy fasta
a0011	0/0	766	1	1	0	0	0	0	EMILIN3_chr20_41354962_41371818	EMILIN3	copy fasta
a0012	0/0	766	1	0	1	0	0	0	EMILIN3_chr20_41354962_41371818	EMILIN3	copy fasta
a0013	0/0	766	1	0	0	1	0	0	EMILIN3_chr20_41354962_41371818	EMILIN3	copy fasta
a0014	0/0	766	1	0	0	1	0	0	EMILIN3_chr20_41354962_41371818	EMILIN3	copy fasta
a0015	0/0	766	1	0	0	0	0	1	EMILIN3_chr20_41354962_41371818	EMILIN3	copy fasta
a0016	0/0	766	1	0	1	0	0	0	EMILIN3_chr20_41354962_41371818	EMILIN3	copy fasta

Continued.

Supplementary Figure 7 | Example of rare haplotype in CHM13v2 human reference assembly while major in GRCh38.

(a) A-level (protein) haplotype diversity for *EMILIN3*, showing 19 distinct A-level haplotypes (a0001–a0019) observed in JoGo 1.0. Amino-acid differences relative to the haplotype of MANE transcript (i.e. GRCh38 coordinate based transcript) is highlighted in green.
(b) Screenshot of the JoGo 1.0 Search by Gene page for *EMILIN3* with the Amino acid (protein) Level Haplotype Info (Total: 19) accordion expanded. The table lists A-level haplotypes (AHAPID) and—by continent (AFR, AMR, EAS, EUR, SAS)—the number of observed haploid chromosomes; light-blue bars visualize these counts. The GRCh38/CHM13v2 column indicates whether the haplotype is present in either reference assembly (codes: 0/0 absent from both; 1/0 present only in GRCh38; 0/1 present only in CHM13v2; 1/1 present in both). The TOTAL column reports the overall number of observations including the references, so it can exceed the sum of the five population counts by up to two. Red arrows highlight the rows for a0001 (major, present in GRCh38; code 1/0) and a0010 (rare, present in CHM13v2; code 0/1). Thus, *EMILIN3* illustrates a case where GRCh38 carries a globally common A-level haplotype (a1), whereas CHM13v2 carries a globally rare one (a10). This major–minor pairing exemplifies how a single reference can encode a haplotype that is uncommon worldwide while JoGo preserves a single global ID ranking that makes such discrepancies immediately apparent.

Supplementary Figure 8

(a)

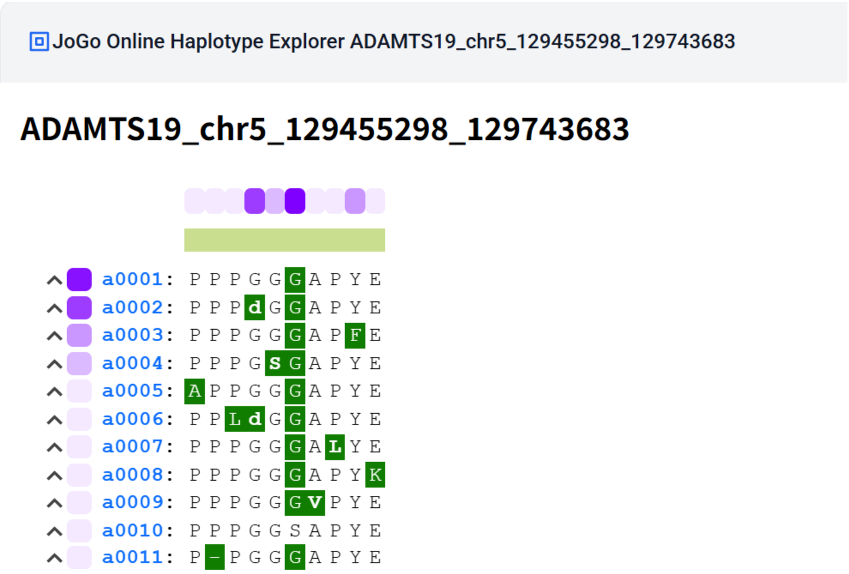

(b)

Search by Gene

Search

JoGo Online Haplotype Explorer ADAMTS19_chr5_129455298_129743683

JoGo Local Haplotype Explorer (on iGV) ADAMTS19_chr5_129455298_129743683

Gene Summary (with MANE)

Translation Multiple Alignment View

LD View

A Amino acid (protein) Level Haplotype Info (Total: 11)

AHAPID	GRCH38/ CHM13V2	ALEN	TOTAL	AFR	AMR	EAS	EUR	SAS	REGIONNAME	GENENAME	AA
a0001	0/1	1213	152	59	26	45	0	21	ADAMTS19_chr5_129455298_129743683	ADAMTS19	copy fasta
a0002	0/0	1215	57	13	6	32	0	6	ADAMTS19_chr5_129455298_129743683	ADAMTS19	copy fasta
a0003	0/0	1213	7	1	5	0	0	1	ADAMTS19_chr5_129455298_129743683	ADAMTS19	copy fasta
a0004	0/0	1213	3	3	0	0	0	0	ADAMTS19_chr5_129455298_129743683	ADAMTS19	copy fasta
a0005	0/0	1213	1	1	0	0	0	0	ADAMTS19_chr5_129455298_129743683	ADAMTS19	copy fasta
a0006	0/0	1215	1	1	0	0	0	0	ADAMTS19_chr5_129455298_129743683	ADAMTS19	copy fasta
a0007	0/0	1213	1	1	0	0	0	0	ADAMTS19_chr5_129455298_129743683	ADAMTS19	copy fasta
a0008	0/0	1213	1	0	1	0	0	0	ADAMTS19_chr5_129455298_129743683	ADAMTS19	copy fasta
a0009	0/0	1213	1	0	0	1	0	0	ADAMTS19_chr5_129455298_129743683	ADAMTS19	copy fasta
a0010	1/0	1213	1	0	0	0	0	0	ADAMTS19_chr5_129455298_129743683	ADAMTS19	copy fasta
a0011	0/0	1209	1	1	0	0	0	0	ADAMTS19_chr5_129455298_129743683	ADAMTS19	copy fasta

Continued.

Supplementary Figure 8 | Example of rare haplotype in GRCh38 human reference assembly while major in CHM13v2.

(a) A-level (protein) haplotype diversity for *ADAMTS19*, showing 11 distinct A-level haplotypes (a0001–a0011) in JoGo 1.0.
(b) JoGo 1.0 Search by Gene snapshot for *ADAMTS19* with the Amino acid (protein) Level Haplotype Info (Total: 11) accordion expanded. As in Supplementary Figure 7b, blue bars encode haploid counts per continent (AFR, AMR, EAS, EUR, SAS). The GRCh38/CHM13v2 codes have the same meaning (0/0, 1/0, 0/1, 1/1). Here, CHM13v2 carries a0001 (code 0/1), which coincides with the globally most frequent A-level haplotype in JoGo 1.0, whereas GRCh38 carries a0010 (code 1/0), which is rare. Notably, the TOTAL for a0010 is 1 even though all five population cells are zero; this reflects a haplotype observed only in the GRCh38 reference and not in our population panel. Together with Supplementary Figure 7, this example demonstrates that either reference can encode minor or even unobserved haplotypes, while JoGo's global ranking makes their rarity explicit and helps users judge whether the reference-encoded haplotype is globally common or rare.

Supplementary Figure 9

(a)

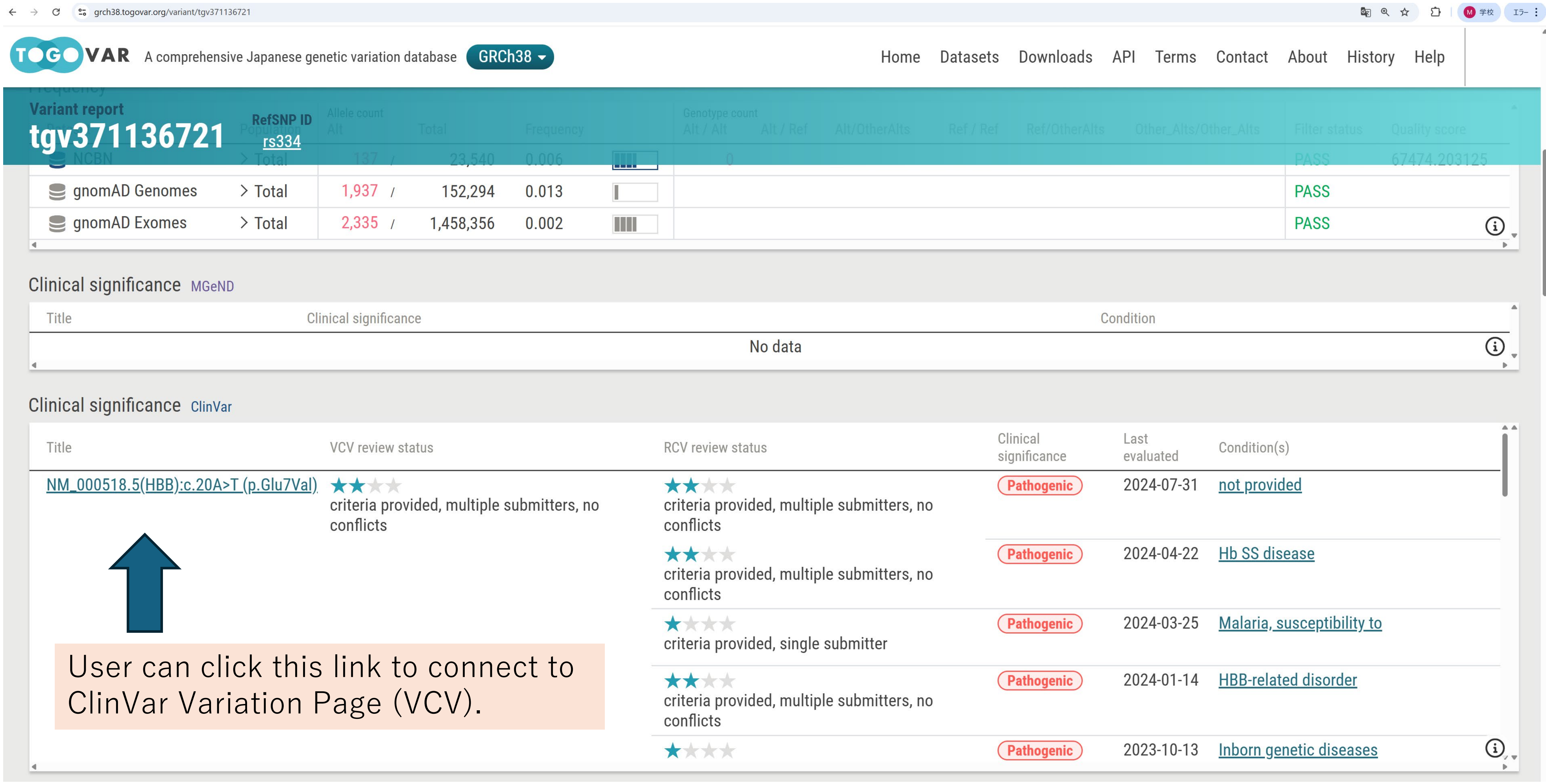

Continued.

(b)

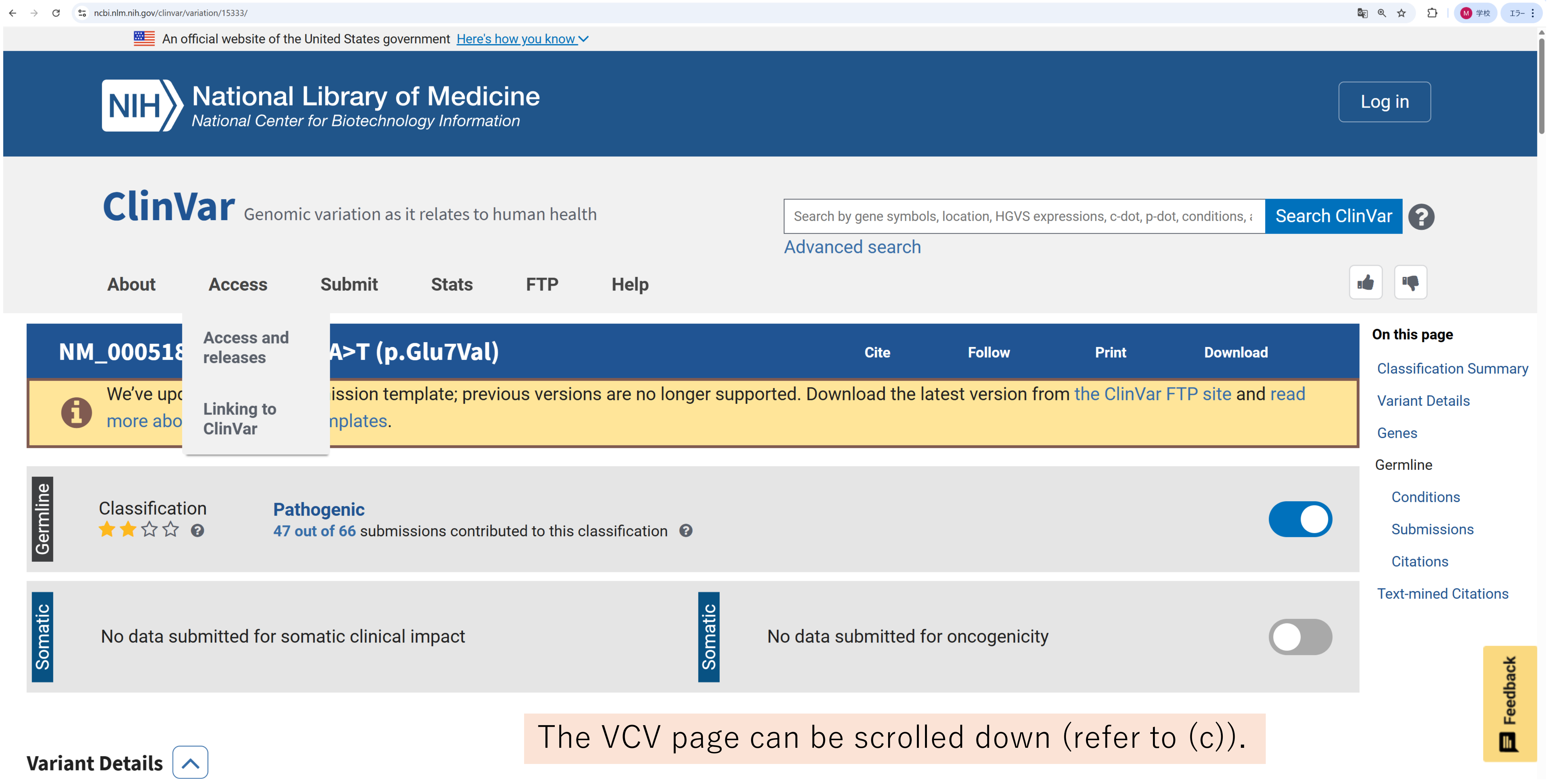
ncbi.nlm.nih.gov/clinvar/variation/15333/
An official website of the United States government Here's how you know
NIH National Library of Medicine
National Center for Biotechnology Information
Log in
ClinVar Genomic variation as it relates to human health
Search by gene symbols, location, HGVS expressions, c-dot, p-dot, conditions,
Search ClinVar
Advanced search
About
Access
Submit
Stats
FTP
Help
Access and releases
Linking to ClinVar
NM_00051
A>T (p.Glu7Val)
Cite
Follow
Print
Download
We've upd
ission template; previous versions are no longer supported. Download the latest version from the ClinVar FTP site and read
more abo
nplates.
On this page
Classification Summary
Variant Details
Genes
Germline
Conditions
Submissions
Citations
Text-mined Citations
Germline
Classification
Pathogenic
47 out of 66 submissions contributed to this classification
Somatic
No data submitted for somatic clinical impact
Somatic
No data submitted for oncogenicity
Feedback
The VCV page can be scrolled down (refer to (c)).
Variant Details

Continued.

(c)

ncbi.nlm.nih.gov/clinvar/variation/15333/

Conditions - Germline

Condition	Classification (# of submissions)	Review status	Last evaluated	Variation/condition record
not provided	Pathogenic (17)	★★☆☆	Jul 1, 2025	RCV000224000.72
HEMOGLOBIN S	not provided (1)	☆☆☆☆	Sep 15, 2023	RCV000016573.22
Hb SS disease	Pathogenic (22)	★★☆☆	Apr 22, 2024	RCV000016574.89
Malaria, resistance to	protective (1)	☆☆☆☆	Dec 2, 2011	RCV000016575.45
beta Thalassemia	Pathogenic (6)	★★☆☆	Oct 18, 2019	RCV000576548.26
Fetal hemoglobin quantitative trait locus 1	Pathogenic (1)	☆☆☆☆	Jul 19, 2018	RCV000723337.12
Dominant beta-thalassemia<br>Erythrocytosis, familial, 6<br>Fetal hemoglobin quantitative trait locus 1<br>Hb SS disease<br>Heinz body anemia<br>METHEMOGLOBINEMIA, BETA TYPE<br>Malaria, susceptibility to<br>alpha Thalassemia<br>beta Thalassemia	Pathogenic (1)	★☆☆☆	Jun 30, 2021	RCV001535873.11
HBB-related disorder	Pathogenic (4)	★★☆☆	Mar 5, 2022	RCV001824571.16
Malaria, susceptibility to	Pathogenic (1)	★☆☆☆	Mar 25, 2024	RCV003989286.3
Beta-thalassemia HBB/LCRB<br>Dominant beta-thalassemia<br>Erythrocytosis, familial, 6<br>Hb SS disease<br>Heinz body anemia<br>Hereditary persistence of fetal hemoglobin<br>METHEMOGLOBINEMIA, BETA TYPE<br>Malaria, susceptibility to	Pathogenic (1)	★☆☆☆	Apr 4, 2024	RCV005049354.1

User can click this link to connect to ClinVar record page (RCV with clinical assertion and evidence).

click to load more conditions

Continued.

(d)

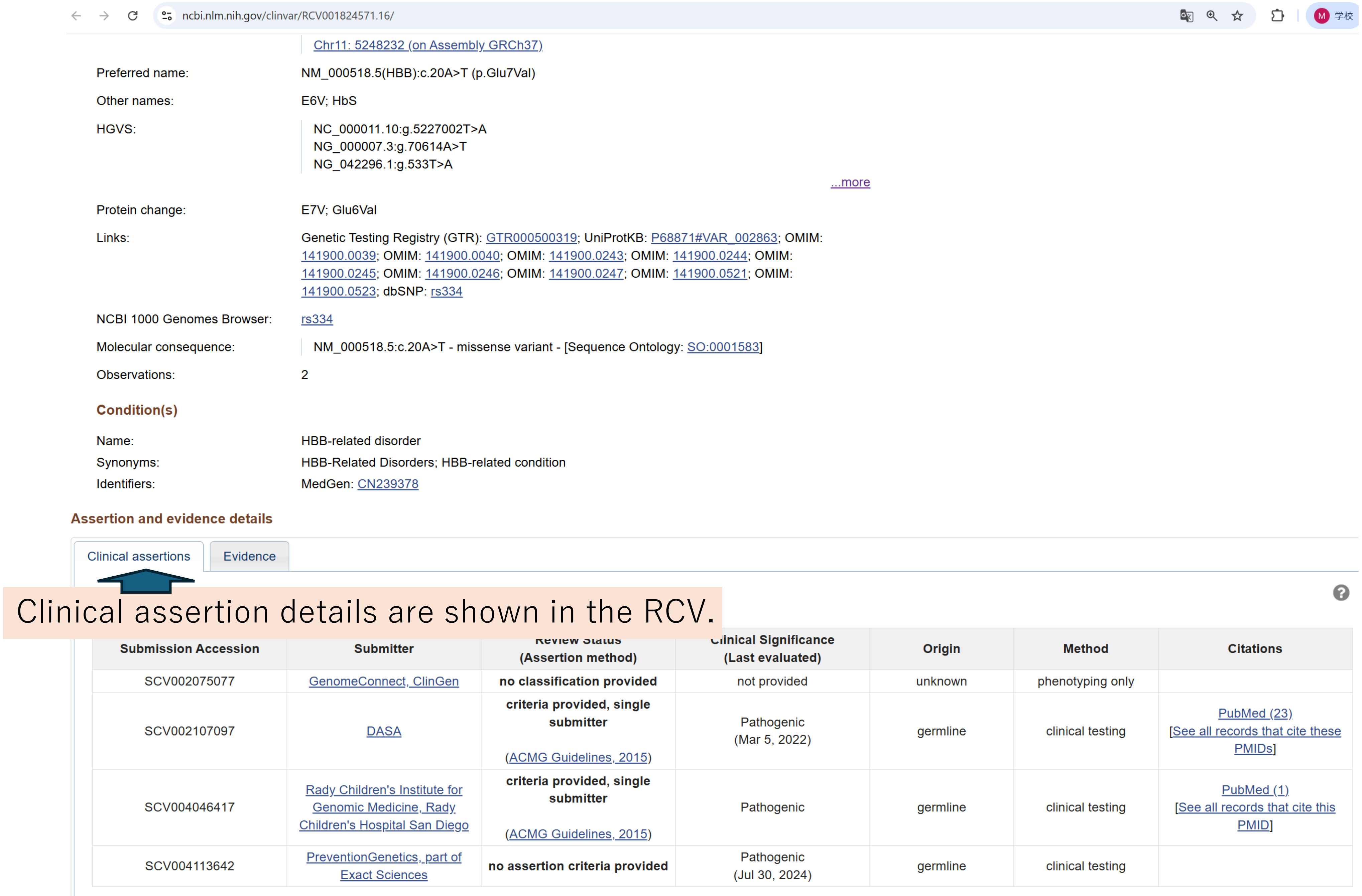

ncbi.nlm.nih.gov/clinvar/RCV001824571.16/

Chr11: 5248232 (on Assembly GRCh37)

Preferred name: NM_000518.5(HBB):c.20A>T (p.Glu7Val)

Other names: E6V; HbS

HGVS: NC_000011.10:g.5227002T>A
NG_000007.3:g.70614A>T
NG_042296.1:g.533T>A
...more

Protein change: E7V; Glu6Val

Links: Genetic Testing Registry (GTR): GTR000500319; UniProtKB: P68871#VAR_002863; OMIM: 141900.0039; OMIM: 141900.0040; OMIM: 141900.0243; OMIM: 141900.0244; OMIM: 141900.0245; OMIM: 141900.0246; OMIM: 141900.0247; OMIM: 141900.0521; OMIM: 141900.0523; dbSNP: rs334

NCBI 1000 Genomes Browser: rs334

Molecular consequence: NM_000518.5:c.20A>T - missense variant - [Sequence Ontology: SO:0001583]

Observations: 2

Condition(s)

Name: HBB-related disorder

Synonyms: HBB-Related Disorders; HBB-related condition

Identifiers: MedGen: CN239378

Assertion and evidence details

Clinical assertions | Evidence

Clinical assertion details are shown in the RCV.

Submission Accession	Submitter	Review Status (Assertion method)	Clinical Significance (Last evaluated)	Origin	Method	Citations
SCV002075077	GenomeConnect, ClinGen	**no classification provided**	not provided	unknown	phenotyping only	
SCV002107097	DASA	**criteria provided, single submitter** (ACMG Guidelines, 2015)	Pathogenic (Mar 5, 2022)	germline	clinical testing	PubMed (23) [See all records that cite these PMIDs]
SCV004046417	Rady Children's Institute for Genomic Medicine, Rady Children's Hospital San Diego	**criteria provided, single submitter** (ACMG Guidelines, 2015)	Pathogenic	germline	clinical testing	PubMed (1) [See all records that cite this PMID]
SCV004113642	PreventionGenetics, part of Exact Sciences	**no assertion criteria provided**	Pathogenic (Jul 30, 2024)	germline	clinical testing	

Continued.

(e)

Assertion and evidence details

Clinical assertions | Evidence

Clinical evidence details are also shown in the RCV

Summary from all submissions

Ethnicity	Origin	Affected	Individuals	Families	Chromosomes tested	Number Tested	Family history	Method
not provided	germline	yes	2	not provided	not provided	not provided	not provided	clinical testing
not provided	germline	unknown	not provided	not provided	not provided	not provided	not provided	clinical testing
not provided	unknown	unknown	3	not provided	not provided	3	not provided	phenotyping only

Citations

PubMed

Gene mutations in human haemoglobin: the chemical difference between normal and sickle cell haemoglobin.
INGRAM VM.
Nature. 1957 Aug 17;180(4581):326-8. No abstract available.
PubMed [citation] PMID: 13464827

Primary role for adherent leukocytes in sickle cell vascular occlusion: a new paradigm.
Turhan A, Weiss LA, Mohandas N, Coller BS, Frenette PS.
Proc Natl Acad Sci U S A. 2002 Mar 5;99(5):3047-51.
PubMed [citation] PMID: 11880644 PMCID: PMC122470

Continued.

Supplementary Figure 9 | End-to-end evidence trail from JoGo (haplotype-level), TogoVar (variant-level), to ClinVar (clinical-level).

Together, panels (a)–(e) demonstrate that users can drill down from haplotype-level patterns (JoGo 1.0) to variant-level evidence (TogoVar → ClinVar: VCV → RCV) and inspect the underlying assertion/evidence without leaving the analytic workflow.
(a) Variant page view after clicking the TogoVar clinical annotation link in the Online Haplotype Explorer tooltip of the HBB haplotype's V character in Figure 3B. The user can select the NM_000518.5(HBB):c.20A>T, p.Glu7Val link to access the ClinVar variation (VCV) page.
(b) VCV page view; the page header summarizes the current germline clinical classification and provides navigation to detailed sections.
(c) Scrolling down the VCV page in (b) shows the "Conditions – Germline" table. This table lists each condition associated with the variant (e.g., Hb SS disease, β-thalassemia, malaria-related entries) together with the condition-specific record accession (RCV). Selecting an RCV opens a page that aggregates submitter-level assertions for that condition.
(d) Example RCV page (e.g., RCV000576548 for β-thalassemia) with the "Assertion and evidence details" panel, which enumerates SCV submissions, assertion methods/criteria (e.g., ACMG), origin/method, and PubMed citations.(e) The "Evidence" tab within the RCV page summarizes clinical evidence across submissions (e.g., affected status, number of individuals/families, testing method), and provides direct links to primary PubMed sources.

Supplementary Figure 10

Iso-Seq Expression profile of 21 tissues with the ACTG-haplotype information on IGV (*HBB* region).

(a)

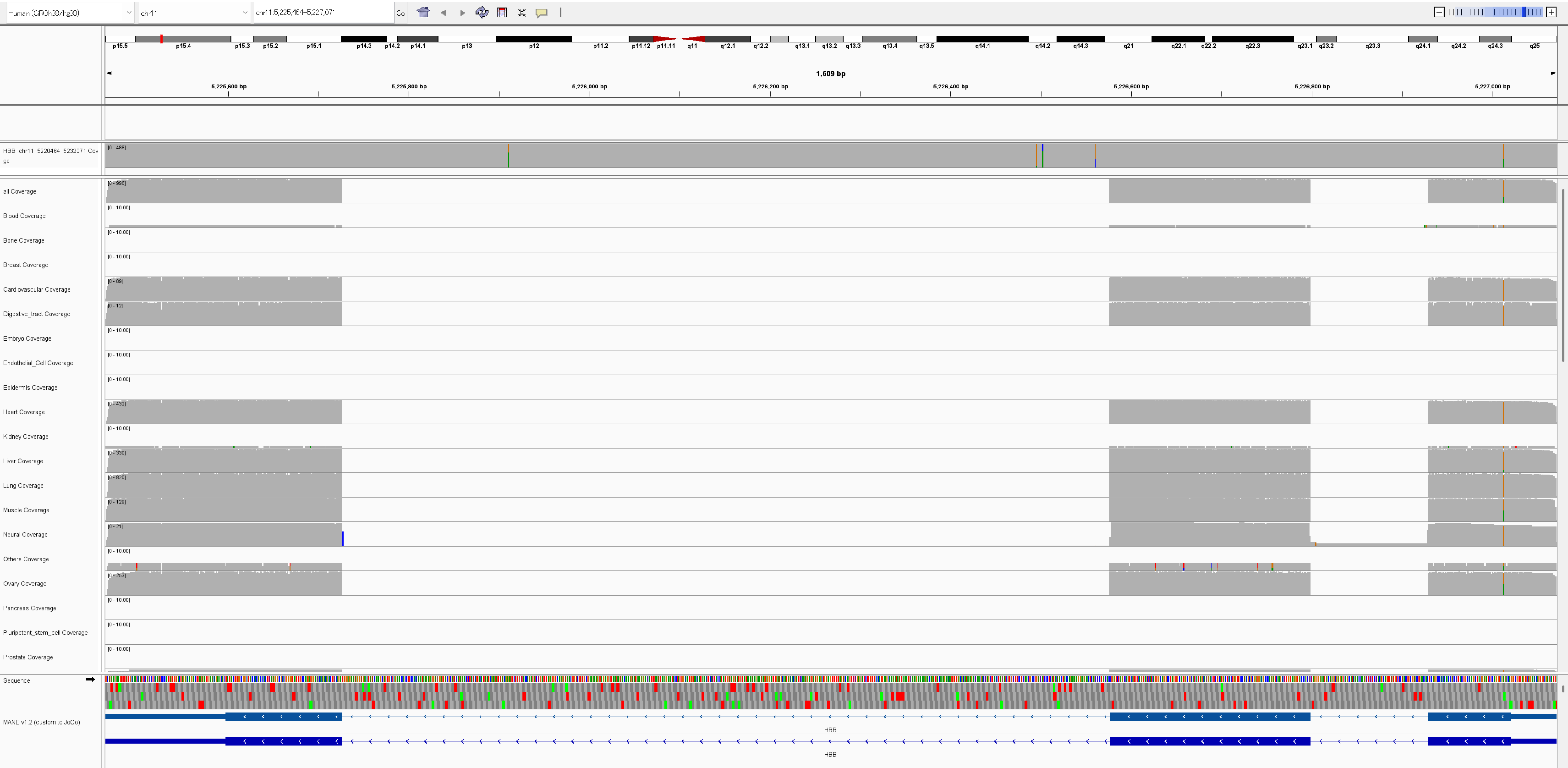

(b)

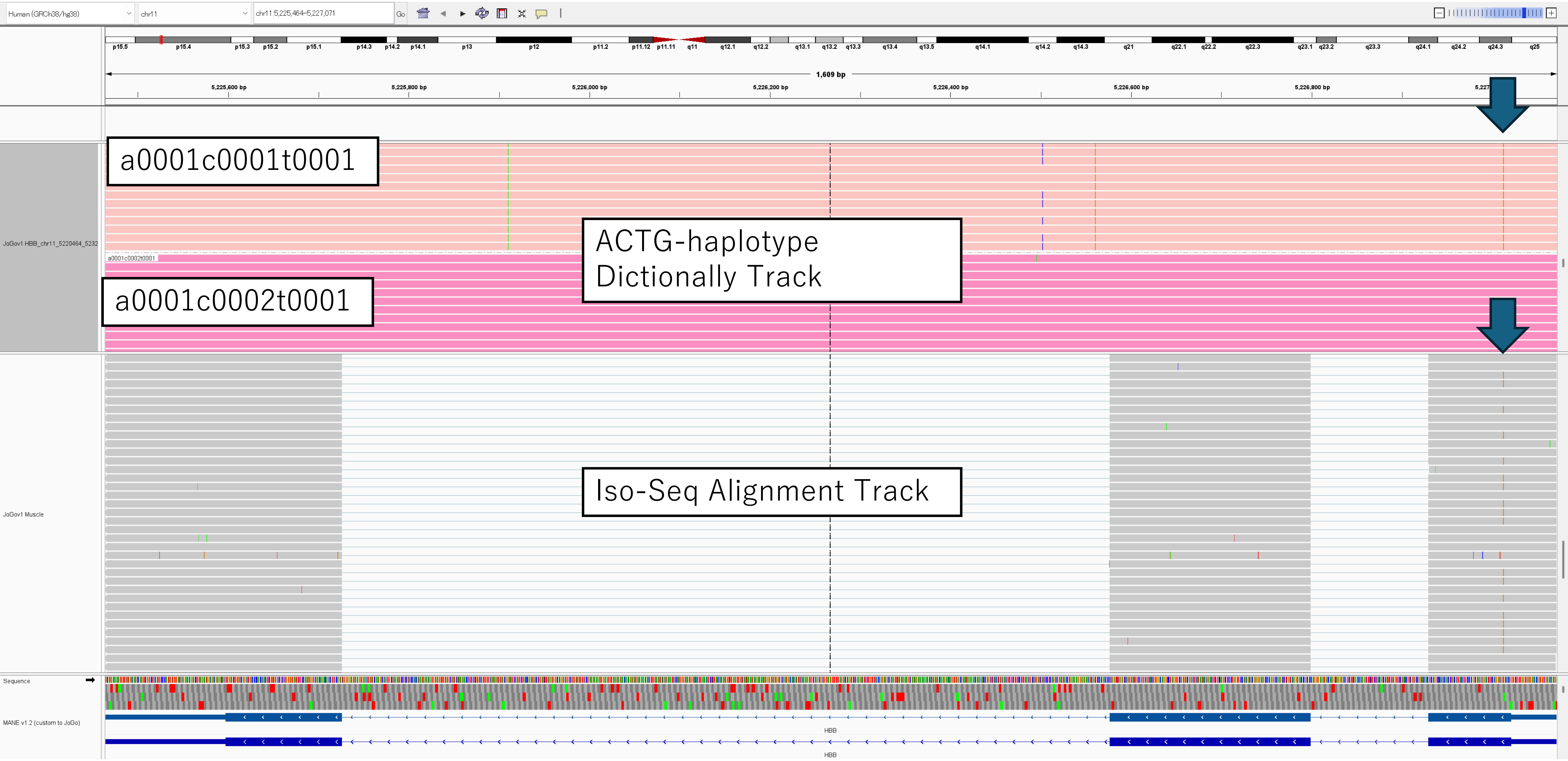

Human (GRCh38/hg38)
chr11
chr11:5,225,464-5,227,071
Go
1,609 bp
5,225,600 bp
5,225,800 bp
5,226,000 bp
5,226,200 bp
5,226,400 bp
5,226,600 bp
5,226,800 bp
a0001c0001t0001
ACTG-haplotype Dictionally Track
a0001c0002t0001
JoGov1 HBB_chr11_5220464_5232
Iso-Seq Alignment Track
JoGov1 Muscle
Sequence
MANE v1.2 (custom to JoGo)
HBB
HBB

Continued.

Supplementary Figure 10 | Iso-Seq expression profile of 21 tissues with the ACTG-haplotype information in IGV (*HBB* region).

(a) IGV session on GRCh38 at the *HBB* locus showing the ACTG-haplotype dictionary/annotation track (top), per-tissue Iso-Seq coverage tracks (middle), and gene models (bottom; MANE v1.2, RefSeq, and CHESS (http://ccb.jhu.edu/chess)).
The coverage panel comprises All Coverage (aggregate of all runs) followed by 21 tissue categories (Supplementary Table 17). Gray bars indicate long-read Iso-Seq coverage; exon–intron structures in the gene model tracks enable visual comparison of haplotype-specific transcript structures and isoform usage within the same genomic window. Together with other public database tracks (GC%, SNPs, repeats), these layers allow users to relate ACTG-haplotypes to tissue-specific expression and splicing directly in a desktop IGV environment.
(b) Base-resolution comparison of ACTG-haplotypes and Iso-Seq reads. The ACTG-haplotype dictionary track (top; JoGo haplotype structure info, grouped and labeled by its concatenated A–C–T ID, e.g., a0001c0001t0001) and the Iso-Seq alignment track (bottom; raw long-read transcript molecules) are both shown in Expanded mode in IGV on the GRCh38 coordinate at the *HBB* locus. Because the two tracks share the same genomic window, single-nucleotide substitutions and small indels that define the dictionary haplotype (colored bases) can be compared directly with those carried by the Iso-Seq reads, allowing users to confirm the expressed haplotype, inspect splice-junction concordance, and assess allele-specific expression or isoform usage. The left bottom arrow indicate the different Iso-Seq read has two haplotype patterns (a0001c0001t0001 and a0001c0002t0001).
