# Supplementary material for: JoGo 1.0: the ACTG hierarchical nomenclature and database covering 4.7 million haplotypes across 19,194 human genes
Source: Nucleic Acids Res. 2025 Nov 29;54(D1):D1159–73. doi: 10.1093/nar/gkaf1232 (PMC12807767; doi:10.1093/nar/gkaf1232)
Supplement: gkaf1232_Supplemental_Files [file gkaf1232_supplemental_files.zip › Supplementary Note.pdf]

Supplementary Note

JoGo 1.0: the ACTG hierarchical nomenclature and database covering 4.7 million haplotypes across 19,194 human genes

Control of Insertion and Deletion Base Quality in ACTG-Haplotype

Insertion and deletion (indel) events within coding regions exert a disproportionate effect on the accuracy of our haplotype catalog. To quantify base-level accuracy in JoGo 1.0, we first measured indel-associated error rates by examining frameshift variants and their local sequencing context. Most of these apparent frameshifts arise from errors in homopolymer runs, a known limitation of long-read technologies. Although such errors rarely prevent correct local diploid assembly, they can introduce spurious frameshifts in coding regions, thereby confounding A- and C-level haplotype definitions.

We therefore implemented a two-step, population-aware correction strategy.

Context-specific indel QV calibration.

We collected all candidate contigs that passed ACTG-haplotype QC, encompassing 5,432,745,246 coding bases (autosomes plus chrX, and chrY). Among these, 101,596 bases were flagged as frameshift sites. We stratified these sites by the length of the homopolymer (separately for A, C, G and T runs) surrounding the variant and—assuming the worst case that every such frameshift is an error—calculated an empirical "per-contig" indel QV for each homopolymer length group (Supplementary Table 4). As expected, these context-specific QVs decline with increasing homopolymer length, and A- or T-runs exhibit slightly higher QVs than C- or G-runs.

Multi-contig Evidence Scaling

Frameshift indels in homopolymer contexts are filtered using a site-specific statistical model that quantifies the probability of observing the supporting reads purely by

sequencing error.
For each candidate variant:

Let N be the number of haplotype-resolved contigs spanning the site.

Let M be the observed allele count (number of contigs carrying the variant).

Let the per-contig indel QV at a candidate site be QV (from the homopolymer table constructed above), corresponding to the per-contig error probability p as

$$p = 10^{-\mathrm{QV}/10}.$$

The number of erroneous variant calls X follows a binomial distribution:

$$\mathrm{X} \sim \mathrm{Binomial}(\mathrm{n} = N, \mathrm{p} = p).$$

The probability of observing at least M erroneous calls is:

$$\mathrm{P}(\mathrm{X} \geq M) = \mathrm{binom.sf}(M - 1, N, p),$$

where binom.sf is the binomial survival function.

This probability is transformed to a variant-level quality value (VQV):

$$\mathrm{VQV} = -10\log10\mathrm{P}(\mathrm{X} \geq M).$$

Threshold

To ensure extremely low false-positive rates in coding sequences, stringent VQV thresholds are applied:

A- and C- level haplotypes: VQV ≥ 100 (error probability ≤ 10^{-10})

T- and G-level haplotypes**:** VQV ≥ 50 (error probability ≤ 10^{-5})

Variants with allele count of 1 are removed outright, as single observations are highly likely to be artifacts.

At VQV 100 ($p = 10^{-10}$), the expected number of erroneous bases among 5,432,745,246 coding positions is

$$5.432745246 \times 10^{9} \times 10^{-10} \approx 0.54 \text{ bases},$$

i.e., effectively zero. After applying this filter, passed frameshift calls passed into the final ACTG-haplotype catalog. This binomial-probability approach incorporates both the empirical error rate and local sequencing depth, ensuring that retained variants have statistically compelling multi-contig support and that the expected number of spurious A- or C-level frameshifts across all coding bases is effectively zero.

For the broader T- and G-level definitions, we apply a more permissive threshold of adjusted-QV 50, reflecting their lower sensitivity to rare indel artifacts.

Example

To satisfy VQV ≥ 100:

For N= 200 contigs, QV = 47 ($p \approx 2 \times 10^{-5}$):

- AC = 1 → removed by the allele-count filter.
- AC = 2 → $P(X \geq 2) \approx 3.98 \times 10^{-8}, VQV \approx 74.0$ → fails the A/C-level threshold (100), removed.
- AC = 4 → $P(X \geq 4) \approx 1.55 \times 10^{-15},\ VQV \approx 148.1$ → passes the threshold.

Definitions for Cover ratio and Completeness ratio

Setup (per gene × ACTG level). We treat each allele observation as an independent haploid sampling unit. For a given gene and level $\ell \in \{A, C, T, G, AC, ACT, ACTG\}$, let the multiset of observed haplotype labels induce an abundance vector whose distinct keys define the observed haplotypes.

Good–Turing coverage (cover ratio)

Definition:

$$C_{GT} = 1 - \widehat{p}_0 = 1 - \frac{f_1}{N},$$

where N is the total number of haploid observations (allele draws), f_1 is the number of

haplotypes observed exactly once (singletons), and $\hat{p}_0$ denotes the Good–Turing estimate of the unseen probability mass. Thus, C_{GT} is the probability that a newly sampled haplotype would match one already registered in JoGo 1.0.

Chao1 richness and completeness (completeness ratio)

Richness estimator (bias-corrected):

$$\hat{S}_{\text{Chao1}} = S_{\text{obs}} + \frac{{f_1}^2}{2\,f_2} \quad (f_2 > 0)$$

$$\hat{S}_{\text{Chao1}} = S_{\text{obs}} + \frac{(f_1)(f_1 - 1)}{2} \quad (f_2 = 0)$$

Completeness ratio:

$$C_{\text{Chao1}} = \frac{S_{\text{obs}}}{\hat{S}_{\text{Chao1}}},$$

where S_obs is the number of distinct haplotypes observed, f1 and f2 are the counts of singletons and doubletons, respectively; $\hat{S}_{\text{Chao1}}$ is the Chao1 lower-bound estimator of the total number of distinct haplotypes. Completeness ratio C_{Chao1} quantifies the fraction already catalogued relative to the predicted total.

Sampling frame and reporting

All metrics were computed per gene × level using the 19,194 genes. We summarize cover ratio and completeness ratio across genes (Supplementary Table 13). The result would be more reliable for 18,095 genes with ≥200 available haploids compared to remaining genes with <200.
